# Sequence features accurately predict genome-wide MeCP2 binding *in vivo*

**DOI:** 10.1038/ncomms11025

**Published:** 2016-03-24

**Authors:** H. Tomas Rube, Wooje Lee, Miroslav Hejna, Huaiyang Chen, Dag H. Yasui, John F. Hess, Janine M. LaSalle, Jun S. Song, Qizhi Gong

**Affiliations:** 1 Carl R. Woese Institute for Genomic Biology, University of Illinois, Urbana-Champaign, Champaign, Illinois 61801, USA; 2 Department of Physics, University of Illinois, Urbana-Champaign, Champaign, Illinois 61801, USA; 3 Department of Biological Sciences, Columbia University, New York, New York 10027, USA; 4 Department of Cell Biology and Human Anatomy, University of California School of Medicine, Davis, California 95616, USA; 5 Division of Life Sciences, Korea University, Seoul 136-713, Korea; 6 Department of Medical Microbiology and Immunology, Genome Center, MIND Institute, University of California School of Medicine, Davis, California 95616, USA; 7 Department of Bioengineering, University of Illinois, Urbana-Champaign, Champaign, Illinois 61801, USA; 8 Department of Biostatistics and Epidemiology, University of California, San Francisco, California 94158, USA

## Abstract

Methyl-CpG binding protein 2 (MeCP2) is critical for proper brain development and expressed at near-histone levels in neurons, but the mechanism of its genomic localization remains poorly understood. Using high-resolution MeCP2-binding data, we show that DNA sequence features alone can predict binding with 88% accuracy. Integrating MeCP2 binding and DNA methylation in a probabilistic graphical model, we demonstrate that previously reported genome-wide association with methylation is in part due to MeCP2's affinity to GC-rich chromatin, a result replicated using published data. Furthermore, MeCP2 co-localizes with nucleosomes. Finally, MeCP2 binding downstream of promoters correlates with increased expression in *Mecp2*-deficient neurons.

## Abstract (web summary)

MeCP2 is critical for proper brain development, and mutations in the gene encoding MeCP2 are responsible for several neurological disorders. Here, the authors show that the previously reported genome-wide preference of MeCP2 to methylated CpGs is in part due to MeCP2's affinity to GC-rich chromatin.

## Graphical abstract

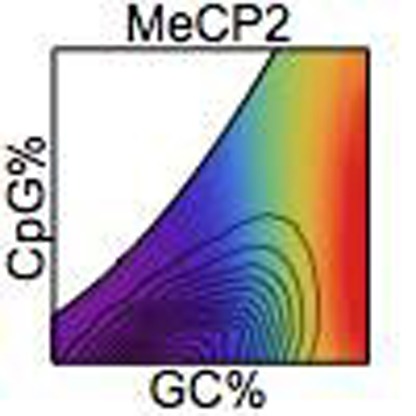

Mutations in the gene encoding methyl-CpG binding protein 2 (*MECP2*) are responsible for several neurological disorders, including the majority of Rett syndrome cases^1^,^2^. Despite extensive efforts since the initial identification of MeCP2 (ref. 3), the molecular mechanisms of its function remain poorly understood. In neurons, MeCP2 is approximately as abundant as histone octamers in the nucleus and is believed to be broadly distributed throughout chromatin^4^. This high abundance has posed a major technical challenge in mapping the genome-wide binding sites of MeCP2 and characterizing the precise DNA sequence features that help recruit MeCP2. Although there is strong evidence *in vitro* supporting the ability of MeCP2 to bind methyl-CpG (mCpG), MeCP2 may actually bind diverse sequences *in vivo*, as reflected in its multifaceted roles^5^,^6^,^7^,^8^,^9^. The functional impact of MeCP2 has been previously examined by attempting to identify MeCP2 target genes in neurons^8^,^10^,^11^,^12^,^13^. In addition to a number of genes found to be suppressed by MeCP2, multiple studies have also identified a global reduction of transcription in neurons lacking functional *Mecp2* (refs 10, 12, 14, 15), suggesting a novel activating role of MeCP2. Resolving whether the resulting repression and activation of genes in *Mecp2*-null neurons are direct or indirect consequences of MeCP2 binding remains a challenge, largely because the resolution of existing chromatin immunoprecipitation sequencing (ChIP-seq) data is not sufficient to decipher the precise binding pattern of MeCP2 *in vivo* and identify the specific DNA recognition sequences^4^,^7^,^16^,^17^.

Using new high-resolution MeCP2 ChIP-seq data from olfactory epithelium, our study presents a predictive model of genome-wide MeCP2-binding pattern. Furthermore, integrative analysis of sequence features and DNA methylation states revealed that the previously reported methylation preferences may arise in part from MeCP2's strong association with GC-rich chromatin, and this surprising result is replicated in independent published data sets. Finally, we describe the impact of *Mecp2* deficiency on transcriptional regulation.

## Results

### High-resolution map of MeCP2 genome-wide localization

**Figure 1:**
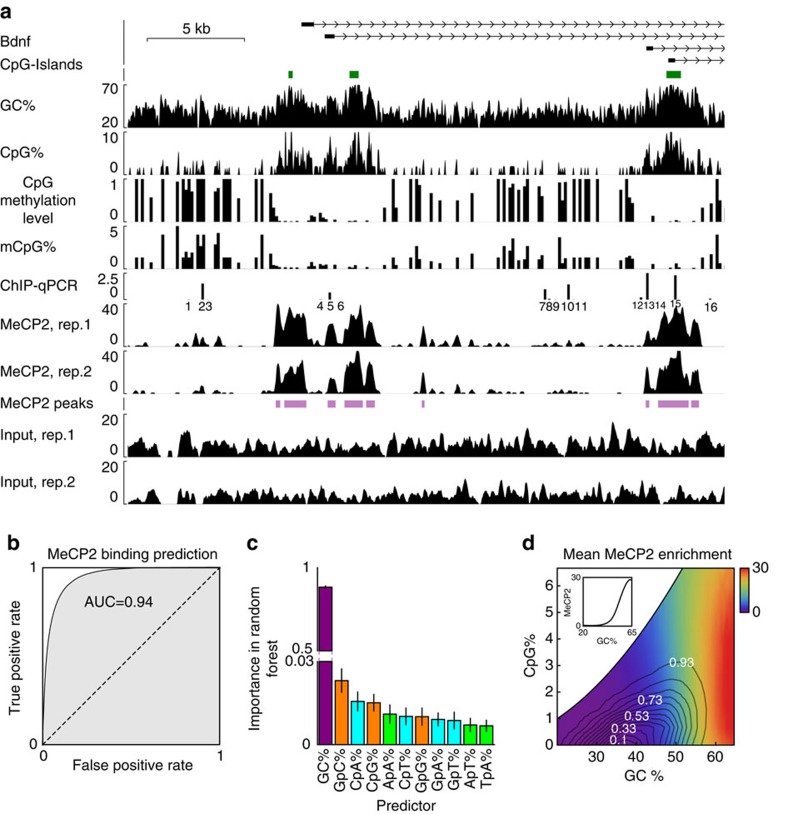
MeCP2 binding is accurately predicted by GC%. (**a**) Wiggle plots showing sequence GC%, CpG methylation level, mCpG% and MeCP2 ChIP-seq and Input profiles for each replicate around the *Bdnf* locus. ChIP-qPCR bars indicate MeCP2 ChIP/Input (%). See Supplementary Table 1 for primer sequences. (**b**) ROC curve for predicting MeCP2 ChIP-seq peaks using Random Forest regressor based on GC% in 200 bp windows. (**c**) Relative importance of different sequence features in predicting MeCP2 binding using the Random Forrest regressor algorithm (Methods). Trees with maximal depth 8 were used, but the dominant importance of GC% did not depend on this choice. (**d**) GC% and CpG% dependence of the mean MeCP2 enrichment (colours) calculated using 150 bp windows. The contours indicate the genome-wide joint distribution *p*(GC%, CpG%) and contour labels indicate the enclosed genome fraction. The inset shows mean MeCP2 enrichment versus GC%.

We overcame the difficulty of mapping genome-wide binding sites of MeCP2 by performing ChIP-seq in olfactory epithelial tissue which contains only one neuronal type, the olfactory sensory neurons^18^. We started with small pilot studies and progressively increased the sequencing depth to reach saturation. Possible chromatin-shearing biases were controlled by using an Input library built from the same pool of chromatin used for ChIP. The final two biological replicates yielded 220 million raw reads from one of the samples sequenced in one lane and 455 million raw reads from the second sample sequenced in two lanes. Eighty-three per cent of the raw reads aligned to the mm9 mouse genome, and 77% of these alignments were unique. PCR duplicates accounted for 53 and 82% of the reads, indicating that saturation was achieved in the final sample. The replicates were then combined in the downstream analysis (Supplementary Fig. 1a–c), obtaining a high depth of sequencing hitherto unavailable for MeCP2. A rigorous normalization using signal extraction scaling^19^,^20^ confirmed the high level of chromatin immunoprecipitation and showed that 29% of the genome was enriched for MeCP2 (Supplementary Fig. 1d). By contrast, reanalysis showed that three other MeCP2 ChIP-seq data sets published to date had low ChIP enrichment, as measured by signal extraction scaling and by the high correlation of ChIP and Input channels (Supplementary Fig. 1e–i). Regions with strong enrichment in our data (that is, statistically significant at 5% false discovery rate) were identified using model-based analysis of ChIP-seq (MACS2)^21^, yielding 1.0 million peaks covering 11% of the genome. Consistent with previous studies^4^, we found that MeCP2 bound the genome at high frequency (average peak spacing of 2.4 kb). The high sequencing depth allowed us to map MeCP2 binding with unprecedented resolution as illustrated for the *Bdnf* locus in Fig. 1a (Myc locus in Supplementary Fig. 2). This map revealed the binding profile to be highly non-uniform on short scales; MeCP2 has sharp peaks with median width 183 bp, and the half width at half maximum of autocorrelation was 113 bp (Supplementary Fig. 1j). ChIP-qPCR (quantitative PCR) around the *Bdnf* gene confirmed that four local peaks and seven local troughs in the ChIP-seq profile indeed had high and low MeCP2 binding, respectively (regions 1–3 and 12–16 in Fig. 1a). We found that three loci (regions 8, 9 and 11) out of five covering an extended region with low ChIP-seq signal had low ChIP-qPCR signal, while remaining two loci (regions 7 and 10) showed moderate ChIP-qPCR enrichment, which might correspond to transient binding missed in ChIP-seq (Fig. 1a). Our new data thus show that MeCP2-binding sites are highly localized at fine resolution.

### MeCP2 peaks are enriched for mCpG

While MeCP2 has a methyl-binding domain that specifically binds mCpG^3^,^22^, it also has domains that can bind non-methylated DNA^7^,^23^. The relative importance of these domains *in vivo* is unclear: some studies reported that MeCP2-binding tracks the density of mCpG^4^, but another study found that the majority of promoters with the highest methylation levels are not bound by MeCP2 (ref. 8). To investigate whether DNA methylation regulates MeCP2 binding in olfactory epithelial tissue, we mapped methylated cytosines using whole-genome bisulfite sequencing in two biological replicates. The CpG methylation levels, defined at each CpG site to be the fraction of cells having methylation at the site, agreed well between the samples (*r*=0.89 in 10 kb windows, Supplementary Fig. 1k), and the replicates were combined for downstream analysis. The methylation level also had strong overlap with previous studies in frontal cortex^24^ and embryonic stem cells (ESC)^25^ (*r*=0.79 and *r*=0.76, respectively, Supplementary Fig. 1l,m). The genome-wide CpG methylation level was 70%. We then calculated the density of methylated CpG dinucleotides (mCpG%) defined as the product of the density of CpG (that is, the percentage of CpG dinucleotides in a 150 bp window, henceforth denoted CpG%) and the local CpG methylation level (that is, CpG methylation level averaged over the same window). Earlier studies found a genome-wide correlation between MeCP2 binding and mCpG%, albeit the resolution was limited^4^,^17^. We also observed that the MeCP2 ChIP-seq coverage was correlated with mCpG% genome wide (*r*=0.39; Supplementary Fig. 3a), while the Input coverage showed no correlation (*r*=0.05; Supplementary Fig. 3b). Strikingly, the mean mCpG% in MeCP2 peaks was 1.7-fold higher compared with adjacent regions (1.26% versus 0.73%), and this increase closely tracked the outline of the peaks (Supplementary Fig. 3c). However, inspection of individual MeCP2 peaks revealed that many binding sites had low mCpG% (Fig. 1a; Supplementary Fig. 2); genome wide, 37% of peaks had lower mCpG% than the surrounding regions (Supplementary Fig. 3d). Thus, while MeCP2 peaks are generally enriched for high mCpG%, the fact that many individual peaks have depleted methylation indicates that other factors are likely to contribute to the binding profile.

### DNA sequence features can predict MeCP2-binding sites

The abundance of MeCP2-binding sites with depleted methylation prompted us to investigate whether the binding pattern of MeCP2 can be predicted from sequence alone. Because most CpGs are methylated in neuronal cells, an enrichment of CpG has been observed in MeCP2-bound regions^4^. Consistent with these results, our data showed coincidence of MeCP2 binding and high CpG% (for example, *Bdnf* locus in Fig. 1a, *Myc* locus in Supplementary Fig. 2). This observation generalized genome wide with a Pearson correlation between MeCP2 ChIP fragment coverage and CpG% of *r*=0.40 (Supplementary Fig. 3e). In contrast to the ChIP signal, Input showed no correlation with CpG% (*r*=−0.03, Supplementary Fig. 3f). The majority of MeCP2 peaks (68%) had higher CpG% than surrounding regions (Supplementary Fig. 3g), and the mean CpG% was significantly higher in the peaks (1.6-fold higher, Wilcoxon rank-sum test, *P*<2.2 × 10^−308^; Supplementary Fig. 3h).

To rigorously characterize MeCP2 recognition motifs in an unbiased way, we trained a Random Forest regressor to predict MeCP2 ChIP-seq peaks purely based on the sequence in 200 bp running windows (see Methods section). Strikingly, mononucleotide frequencies alone had very high predictive power (area under receiver-operating characteristic (ROC) curve (AUC), of 94%; Fig. 1b). Inclusion of di- and trinucleotides did not increase the predictive power appreciably (AUC of 95% for trinucleotides), but a model using only CpG% had much lower predictive power (AUC of 78%; Supplementary Fig. 3l). Equating false positive and negative rates on the ROC curve yielded a classifier with 88% accuracy. In sharp contrast to the previously reported CpG preference^4^, ranking the mono- and dinucleotides by their predictive importance showed that GC% was by far the most important feature, while CpG% was only ranked the 4th out of 11 (Fig. 1c). Our analysis thus suggests that GC%, and not CpG%, is an accurate predictor of MeCP2 binding. Indeed, the genome-wide Pearson correlation of the MeCP2 fragment coverage with GC% was higher than that with CpG% (*r*=0.68 versus *r*=0.40, Supplementary Fig. 3e,f,i,j), and 98% of peaks had significantly higher GC% than surrounding regions (Wilcoxon rank-sum test *P*<2.2 × 10^−308^; Supplementary Fig. 3k).

**Figure 2:**
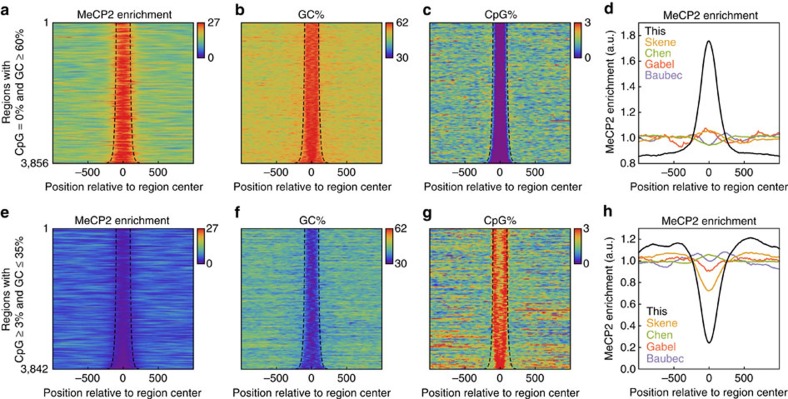
MeCP2 binding follows GC% in regions with extreme GC% and CpG%. (**a**–**c**) Alignment plots showing MeCP2 enrichment, GC%, and CpG% (colour) around regions with no CpG and GC≥60%. These regions were identified genome wide using sliding windows, and overlapping regions were joined (the resulting regions are outlined by dashed curves). (**d**) Comparison of the aligned MeCP2 ChIP-seq signal in this and previously published studies using the regions in **a**–**c**. For Baubec *et al*., the figure shows the ESC data. The *y* axis represents ChIP/Input for all data sets, except for Skene *et al*. which does not have Input. (**e**–**g**) Same as **a–c** but showing regions with CpG≥3% and GC≤35%. (**h**) Same as **d** but using the regions from **e**–**g**.

To reconcile the high enrichment of CpG in MeCP2 peaks with the low importance of CpG% as a binding predictor, we controlled for the genome-wide correlation between CpG% and GC% by first classifying 150 bp genomic windows by GC% and CpG% and then calculating how the mean MeCP2 coverage depended on these two variables. This representation clearly revealed that the coverage depended strongly on GC% but not on CpG% (Fig. 1d; Supplementary Fig. 4a,b). Most of the correlation between CpG% and MeCP2 signal was removed once controlled for GC dependence (partial correlation *r*_MeCP2,CpG·GC_=0.12 compared with *r*_MeCP2,CpG_=0.40), but the reverse control (that is, controlling for CpG dependence in the correlation between GC% and MeCP2) barely reduced the correlation (*r*_MeCP2,GC·CpG_=0.61 compared with *r*_MeCP2,GC_=0.68). Partial correlation analysis thus effectively removes secondary correlations arising from confounding factors, and our ensuing analyses extensively utilize and generalize this approach. The secondary association between MeCP2 and CpG% was further supported through reanalysis of previous MeCP2 ChIP-seq data from mature murine neurons^4^ (*r*_MeCP2,GC·CpG_=0.34 and *r*_MeCP2,CpG·GC_=0.02; Supplementary Fig. 4c). Reanalysis of the data from ES cells and ES-derived neuronal progenitor cells revealed a somewhat stronger CpG dependence (neuronal progenitor, *r*_MeCP2,GC·CpG_=0.30, *r*_MeCP2,CpG·GC_=0.14; Supplementary Fig. 4d,e)^17^, but ES cells lacking the methyltransferases *Dnmt1, Dnmt3a* and *Dnmt3b* had very weak CpG dependence but still an appreciable GC dependence (*r*_MeCP2,GC·CpG_=0.20, *r*_MeCP2,CpG·GC_=0.02; Supplementary Fig. 4f). The weaker CpG dependence in the more differentiated cells is potentially explained by the large increase in MeCP2 expression throughout neural differentiation^26^ and a shift from methylation-dependent to methylation-independent binding in the high free-protein concentration limit. Reanalysis of published data from murine forebrain and hypothalamus revealed enrichment profiles constant in both GC% and CpG% (Supplementary Fig. 4g,h)^9^,^27^, a different pattern potentially explained by the much lower ChIP versus Input enrichment seen in these data sets (Supplementary Fig. 1e) or, for hypothalamus, low coverage. To verify that the above genome-wide binding trends were visible also at individual loci, we plotted the ChIP signal in short regions with contrasting GC% and CpG%. Strikingly, regions with no CpG but GC%≥60% had strong MeCP2 enrichment, supporting that CpG is not necessary for MeCP2 binding (Fig. 2a–c); conversely, regions with CpG%≥3% but GC%≤35% showed decreased MeCP2 enrichment (Fig. 2e–g). This binding pattern was further corroborated by three independent lower-coverage ChIP-seq replicates from the pilot stage of our study (Supplementary Fig. 4i–n). However, although some previously published data sets also had a moderate enrichment and depletion in high and low GC% regions, respectively, the trends were less pronounced compared with olfactory epithelial tissue (Fig. 2d,h). To control for the possibility that the signal was caused by potential GC bias in the sequencing adapter ligation or library amplification, we also performed ChIP-qPCR in nine regions with high GC% and no CpG and 13 regions with low GC% but high CpG% and again found that the binding follows GC% and not CpG% (Supplementary Fig. 4o; Supplementary Table 2). Modest correlation of MeCP2-binding strength with GC% could be also seen *in vitro* by electrophoretic mobility shift assay (EMSA) (Supplementary Fig. 5b,c; Supplementary Table 3; Supplementary Methods). Taken together, these findings show that the primary sequence predictor of MeCP2 binding is GC% and that the previously reported correlation between CpG density and MeCP2 enrichment is mostly explained by the confounding correlation of GC% with CpG%.

### Controlling for GC% reduces mCpG dependence of binding

**Figure 3:**
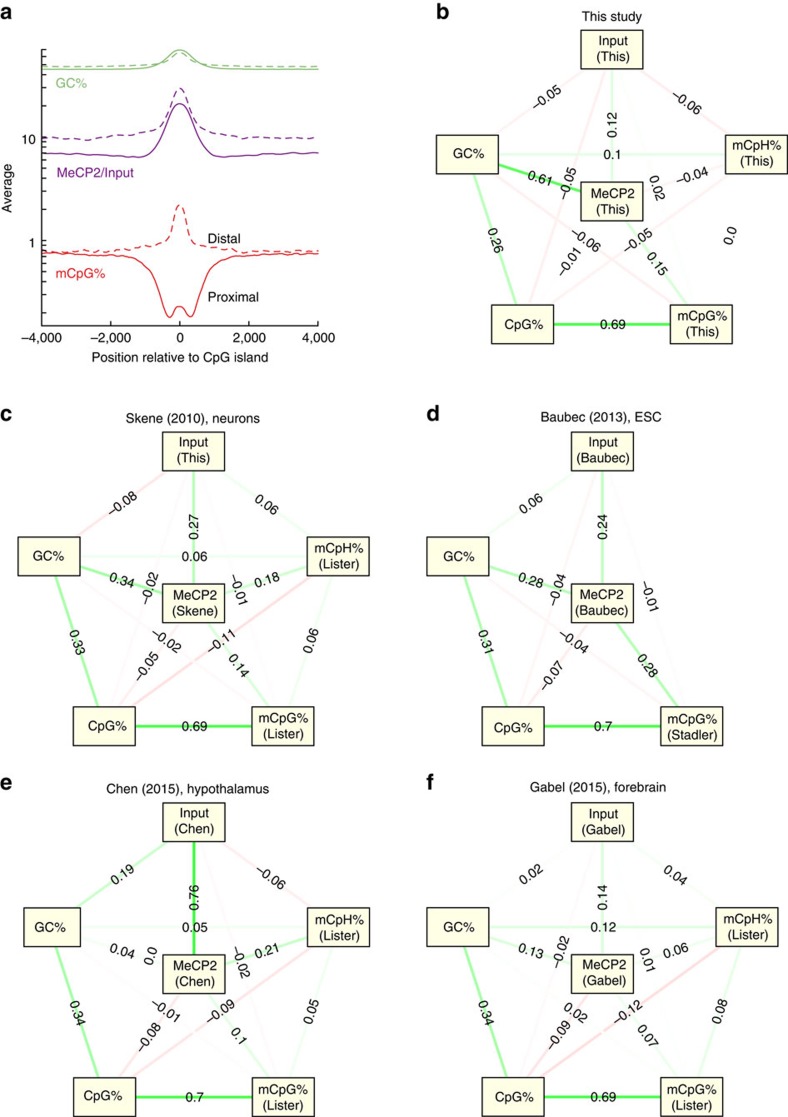
MeCP2 enrichment depends weakly on mCpG%. (**a**) GC% (green), mean MeCP2 enrichment (purple) and mCpG% (red) around proximal (that is, located within 500 bp of a TSS, solid) and distal (dashed) CGIs. (**b**) Gaussian graphical model (Methods) describing the full partial correlations between the MeCP2 ChIP, Input, GC%, CpG%, mCpG% and mCpH%, all evaluated using 150 bp binning. (**c**) Same as **b** but using MeCP2 ChIP-seq data from Skene *et al*.^4^ (no Input was available) and methylation data from Lister *et al*.^24^ (**d**) Same as **b** but using ES cell MeCP2 ChIP and Input from Baubec *et al*.^17^ and methylation data from Stadler *et al*.^25^ (no mCpH% was available). (**e**) Same as **b** but using MeCP2 ChIP and Input from Chen *et al*.^27^ and methylation data from Lister *et al*. (**f**) Same as **b** but using MeCP2 ChIP and Input from Gabel *et al*.^9^ and methylation data from Lister *et al*.^24^

Even though methylated CpG clearly increased the binding affinity of MeCP2 *in vitro* (Supplementary Fig. 5d,e; Supplementary Table 3)^28^,^29^, the corresponding *in vivo* association assessed by MeCP2 ChIP-seq was only moderate. We further investigated the extent to which the GC% dependence confounded the association between MeCP2 and mCpG% and found that much of the association was removed after controlling for GC% (*r*_MeCP2,mCpG_=0.39, *r*_MeCP2,mCpG·GC_=0.19, 150 bp windows; Supplementary Fig. 6a). In contrast, the MeCP2 enrichment depended strongly on GC% even after controlling for mCpG% (*r*_MeCP2,GC·mCpG_=0.62). Reanalysis of published MeCP2 ChIP-seq and cell type-matched bisulfite sequencing data revealed a similar dichotomy in the brain (*r*_MeCP2,mCpG·GC_=0.13 versus *r*_MeCP2,GC·mCpG_ =0.34; Supplementary Fig. 6b)^4^,^24^. The MeCP2 binding in ES cells had a stronger dependence on mCpG% (*r*_MeCP2,mCpG·GC_=0.32, *r*_MeCP2,GC·mCpG_=0.29; Supplementary Fig. 6c)^17^,^25^. As in the above CpG% analysis, the published data from murine forebrain and hypothalamus were uniform in mCpG% and GC% (Supplementary Fig. 6d,e)^9^,^27^. The predictive power of GC% was particularly clear in CpG islands (CGIs): in CGIs proximal to promoters (within 500 bp of the transcription start sites (TSS)), mCpG% was suppressed relative to surrounding regions, but both GC% and MeCP2 signal were distinctly elevated (Fig. 3a). Contrasting this, distal CGIs were enriched for mCpG%, GC% and MeCP2 binding (Fig. 3a). Thus, while MeCP2-binding sites are enriched for both high GC% and mCpG%, the binding profile generally follows GC%, even where GC% and mCpG% diverge.

A recent study argued that MeCP2 also recognizes methylated cytosine in the mCpH context (H=A,T,C)^27^. To systematically investigate the importance of mCpH%, we next built a Gaussian graphical model for MeCP2 binding and explanatory covariates. Briefly, a Gaussian graphical model represents the conditional dependence of random variables as an undirected graph, where the nodes are the variables of interest and an edge between two nodes captures the correlation between the connected nodes after controlling for confounding correlations with the remaining nodes. We first built a model relating the MeCP2 ChIP, Input, GC%, CpG%, mCpG% and mCpH% at 150 bp resolution. Consistent with the above observations, we found a chain of strong edges from MeCP2 to GC%, CpG% and finally mCpG% (Fig. 3b). By contrast, the direct edges from MeCP2 to CpG% and mCpG% were both relatively weak, implying that MeCP2 binding was mostly independent of these two sequence features once conditioned on the remaining features. Similarly, mCpH% was very weakly connected to MeCP2 and slightly more correlated with Input, suggesting that mCpH% was a subdominant predictor of MeCP2 binding in olfactory epithelia. Reanalysis of data from neurons and ES cells revealed similar networks, but these data sets showed stronger correlation between MeCP2 ChIP and Input, and the link between MeCP2 and GC% was also somewhat weaker (Fig. 3c,d). The hypothalamus data set showed a stronger edge between MeCP2 ChIP and mCpH% than between MeCP2 and GC% (Fig. 3e), but that data set had a very strong correlation between MeCP2 and Input, making it difficult to interpret the result. Because one data set had very low counts at 150 bp resolution (Fig. 3f), we repeated the analysis at 10 kb resolution and found graphs with similar structures, the main difference being strong correlations between MeCP2 and Input in ESC, hypothalamus and forebrain (Supplementary Fig. 6f–j). Our integrative analysis thus shows that GC% and, to a lesser extent, mCpG% are the main predictors of MeCP2 binding.

### MeCP2 preferentially binds nucleosomal DNA

**Figure 4:**
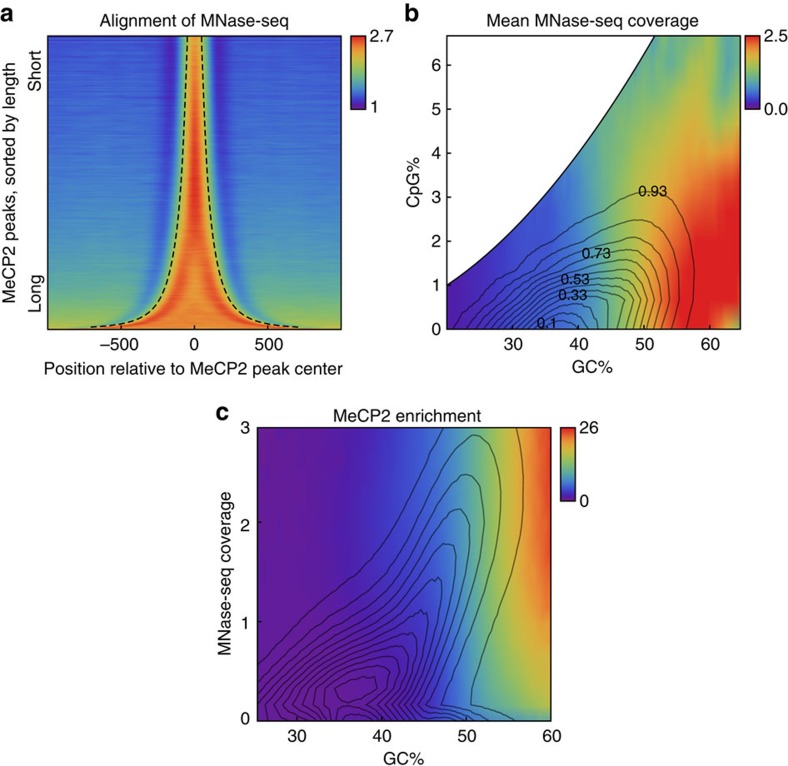
MeCP2 binding coincides with nucleosomes. (**a**) Enrichment plot of WT MNase-seq profile (colour) around MeCP2 ChIP-seq peaks (sorted by length and outlined by dashed curves). (**b**) GC% and CpG% dependence of the mean WT MNase-seq coverage (colours). The contours indicate the genome-wide joint distribution *p*(GC%, CpG%). (**c**) MeCP2 enrichment as a function of GC% and MNase-seq fragment coverage. The contours indicate the genome-wide joint distribution *p*(GC%,MNase-seq).

Given the role of MeCP2 as an epigenetic regulator, we next investigated the interplay between MeCP2 and chromatin structure. Previous *in vitro* studies of MeCP2 found that the C-terminal portion of the protein contains a chromatin-binding domain that facilitates complex formation with nucleosomes^5^. Much like the H1 histone, MeCP2 binds reconstituted nucleosomes near the DNA entry and exit sites and protects proximal linker DNA from digestion^5^,^30^, an observation later corroborated with data obtained from HeLa S3 cells^29^. To investigate this association *in vivo*, we measured the genomic locations of nucleosomes in wild-type (WT) tissue using MNase-seq. The nucleosomes exhibited previously reported patterns: expressed genes (see below) had deep nucleosome-depleted regions around the TSS and distinct +1 nucleosomes (Supplementary Fig. 7a)^31^,^32^. Likewise, the transcription termination sites were also depleted of nucleosomes (Supplementary Fig. 7b). Consistent with a recent study^27^, overlaying the MNase-seq and MeCP2 ChIP-seq data showed preferential co-localization of MeCP2 peaks with nucleosomes (for example, Myc locus in Supplementary Fig. 2). Genome-wide, MeCP2 peaks closely overlapped with sharply increased nucleosome read density (Fig. 4a). Furthermore, the MeCP2 ChIP-seq and MNase-seq fragment densities were highly correlated (*r*=0.66). Finally, the genome-wide cross-correlation between the MeCP2 ChIP-seq and MNase-seq signals was sharply peaked at zero offset (Supplementary Fig. 7c; Supplementary Methods). These pieces of evidence together show that MeCP2 and nucleosomes coincide genome wide.

Like MeCP2 binding, nucleosome occupancy is guided by sequence; previous studies in *Saccharomyces cerevisiae* found that the nucleosome occupancy correlates strongly with GC% (refs 33, 34), and subsequent studies further argued that GC% is the primary predictor of intrinsic nucleosome occupancy^35^. Consistent with these results, we found that the nucleosome occupancy correlates strongly with GC% but not with CpG% (Fig. 4b, *r*_MNase,GC·CpG_= 0.57 >> *r*_MNase,CpG·GC_=−0.12). This sequence dependence is similar to that of MeCP2, and we therefore augmented our previous Gaussian graphical model by including the MNase-seq data. This modification reduced the direct correlation between MeCP2 and GC% from 0.61 to 0.39 and introduced a three-way interaction involving nucleosome occupancy (Supplementary Fig. 7d). Furthermore, at each fixed GC%, plotting the conditional MeCP2 enrichment as a function of MNase-seq density confirmed the dependence of MeCP2 enrichment on nucleosome occupancy (Supplementary Fig. 7e). To investigate the relative effects of nucleosome and GC%, we plotted how the mean MeCP2 enrichment jointly depends on these covariates and found that the GC% dependence was much stronger than the nucleosome dependence (Fig. 4c). Thus, while MeCP2 binding still conditionally depends on nucleosome occupancy when controlled for GC%, its marginal dependence on GC% seems stronger.

### MeCP2-binding pattern correlates with repressor function

The regulatory function of MeCP2 remains unclear. While earlier studies found that MeCP2 acts as a repressor in individual methylated promoters^36^,^37^, subsequent studies did not find a genome-wide increase in gene expression in *Mecp2*-deficient neurons^38^. On the contrary, several recent studies found numerous transcriptional changes in *Mecp2*-deficient tissues, most of which seemed to implicate MeCP2 acting as a global activator^10^,^12^. A previous study reported that genes with increased mCpG% around the TSS exhibited increased MeCP2 binding^17^. Even though we also observed increased MeCP2 binding, GC% and CpG% immediately downstream of the TSS (Supplementary Fig. 8a–c), we found that mCpG% and the CpG methylation level actually decreased around the TSS (Supplementary Fig. 8d,e). Furthermore, the genes with the highest CpG methylation level had the lowest MeCP2 enrichment (Supplementary Fig. 8e). To understand this anticorrelation, we sorted the promoters into quintiles of GC% and CpG methylation level and found that genes with high GC% (and CpG%) had decreased methylation level and mCpG% (as expected given the hypomethylation of proximal CGIs discussed above) and that the anticorrelation between MeCP2 binding downstream of the TSS and CpG methylation level disappeared after conditioning on GC% (Supplementary Fig. 8e). Thus, as was the case in genome-wide analysis, MeCP2 enrichment primarily tracked GC% across the promoter.

To investigate *Mecp2* function in the regulation of transcription genome wide, we performed RNA-seq and compared the WT with *Mecp2* KO. The expression values of the biological triplicates clustered correctly into separate WT and KO groups (Supplementary Fig. 9a). An earlier study found that the majority of MeCP2-bound promoters are actually transcriptionally active, suggesting that even if MeCP2 acts as a repressor, its binding may not completely silence gene expression^8^. To investigate the relation between MeCP2 binding and gene expression, we sorted genes by their expression levels in WT animals and aligned the MeCP2 ChIP enrichment around the TSSs (Supplementary Fig. 9b). While MeCP2 is strongly enriched downstream of the TSS, this enrichment did not correlate with expression status and was almost constant for FPKM values between 0.1 and 50 (Supplementary Fig. 9c,d). In the region upstream of the TSS, the MeCP2 ChIP enrichment was marginally lower in highly expressed genes compared with lowly expressed genes, particularly in the proximal promoter (Supplementary Fig. 9e).

**Figure 5:**
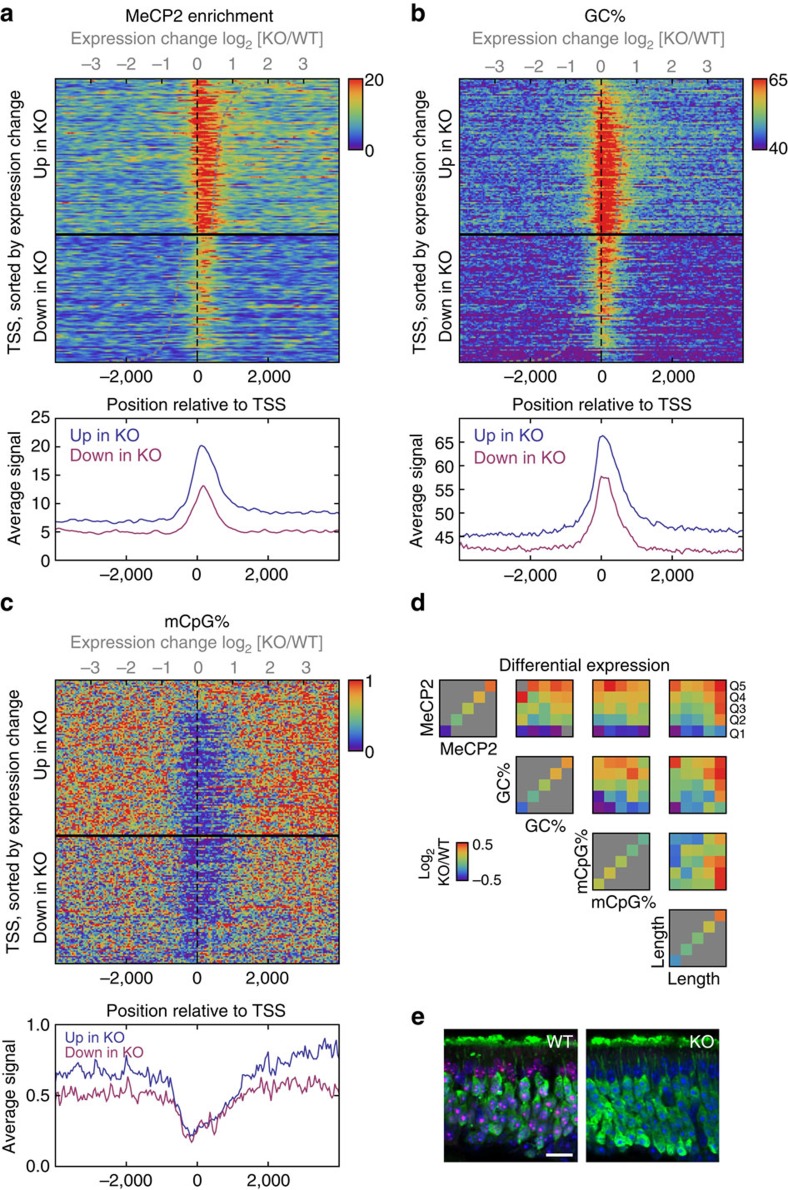
MeCP2 binding at the 5′ end of genes is associated with increased transcription in *Mecp2* KO. (**a**) Top plot shows the alignment of MeCP2 enrichment profiles (colour) in 8 kb regions surrounding the significantly up- and down-regulated genes after *Mecp2* KO (separated by thick horizontal black line). The TSSs are ordered by fold change in expression between KO and WT (indicated by curved grey line). The bottom plot shows the mean MeCP2 enrichment in up- and down-regulated genes. (**b**) Same as **a** but showing GC%. (**c**) Same as **a** but showing mCpG%. (**d**) Dependence of the differential expression on MeCP2 enrichment, GC%, mCpG% and gene length. Each subplot shows how the mean log_2_-fold expression change (colour) depends on a pair of covariates (averaged across the first 500 bps downstream of TSS) after grouping by quintile (columns and rows in subplots). Only genes with significant differential expression are included. Grey indicates combinations without data. The direction of the colour gradient indicates the strongest dependence of the differential expression. (**e**) Olfactory sensory neurons were identified by the expression of olfactory marker protein (OMP, green) and the nuclei were identified by DAPI staining (blue). No significant changes in cell body and nuclei sizes were observed between wild-type (identified by MeCP2 expression (Red), left panel) and KO (shown by lack of MeCP2 expression, right panel). Scale bar, 20 μm. Quantification is shown in Supplementary Fig. 10.

To test whether MeCP2 binding is associated with repressed transcription, we first identified transcripts with significant differences in expression between WT and *Mecp2* KO, giving 1,690 transcripts in total (Supplementary Fig. 9f). The median changes in up- and down-regulated genes were 1.50- and 0.64-fold, respectively. We found that the MeCP2 enrichment downstream of the TSS was 78% higher in upregulated transcripts compared with downregulated transcripts after *Mecp2* KO (Wilcoxon rank-sum test *P*=1.4 × 10^−50^; Fig. 5a; Supplementary Fig. 9g). It should be noted, however, that peak heights might represent either more binding across cell population or more stable binding in a subset of cells; thus, the observed higher MeCP2 enrichment should be interpreted with caution. The GC% in the 5′ end of *Mecp2* KO upregulated genes was also markedly higher than in downregulated genes (65 versus 55%, Wilcoxon rank-sum test *P*=3.7 × 10^−50^), and the increase largely tracked the MeCP2 enrichment in extent and magnitude (Fig. 5b). Although mCpG% did not differ appreciably between the 5′ ends of up- and down-regulated genes (Fig. 5c), MeCP2-binding level did correlate with differential expression (Fig. 5d). Importantly, the differential expression did not depend on GC% or mCpG% after conditioning on MeCP2-binding level (*P*=0.16 and *P*=0.28, regression slope *t*-test on ranked data), suggesting that the dependence of differential expression on MeCP2 was not an indirect effect mediated through GC% or mCpG% (Fig. 5d). Consistent with earlier observations^9^,^39^, long genes were more upregulated than short genes. Furthermore, this length dependence remained after controlling for MeCP2 binding in the promoter, suggesting that it is an effect independent of MeCP2-binding downstream of promoter (Fig. 5d). Taken together, these findings suggest that MeCP2 both directly downregulates genes with strong binding and indirectly downregulates long genes.

Recent studies showed that *Mecp2*-deficient embryonic stem cell-derived neuronal nuclei were smaller in size, had decreased total RNA and rRNA and had down-regulation of both transcription- and translation-related genes^14^,^15^. In contrast to these studies, we did not observe significant differences in either neuronal cell body or nucleus sizes within neuronal epithelium (*t*-test; *P*<0.1, Fig. 5e; Supplementary Fig. 10a,b). *Mecp2* KO mice are often under-nourished and under-weight, but the WT and KO mice used in our study had similar body weight, within 2 g of difference. Of note, cells within the olfactory epithelium are tightly packed, possibly making the detection of subtle differences difficult. However, we observed a 25% reduction in the total RNA extracted from equal number of viable cells from WT and KO (Supplementary Fig. 10c,d), and gene ontology analysis revealed that translation and RNA-processing categories were significantly downregulated (Supplementary Fig. 9h). Furthermore, differentially expressed ribosomal genes, all of which were downregulated, had lower MeCP2 binding than significantly upregulated genes (*t*-test; *P*=1.6 × 10^−4^), suggesting that the observed reduction in transcripts may be due to global alterations in chromatin rather than a direct consequence of local MeCP2 binding at those targets.

## Discussion

This study shows that MeCP2 binds distinct but numerous sites throughout the genome in a manner that can be accurately predicted using DNA sequence features alone. To date, the lack of a fine-resolution genome-wide binding map has been a major bottleneck in understanding the mechanism of MeCP2 function^40^. One challenge has been the fact that MeCP2 has a widespread binding pattern along the entire genome^4^, thereby diluting the ChIP-seq signal when sequenced at shallow depth. Another complication arises from the diversity of neuronal types present in the brain tissue^4^,^27^. In addition to diverse neuronal populations, a large number of glial cells might also reduce the MeCP2 ChIP enrichment level^41^. We have overcome these difficulties by performing high-resolution MeCP2 ChIP-seq in olfactory epithelium, which has only one type of neurons, namely the olfactory sensory neuron. The olfactory epithelial tissue harvested for our experiments consisted of mostly olfactory sensory neurons (77.2%) and only a small fraction of sustentacular cells (14.0%), and underlining stromal cells (8.8%) (data from this study). As a result, distinct individual MeCP2-binding sites could be identified, and only very low correlation was observed between MeCP2 ChIP-seq and Input data, distinguishing our results from previous published data sets^9^,^27^. We believe the preponderance of a unique neuronal population in conjunction with an unconventional deep-sequencing strategy made our high-resolution data possible.

While our data exhibited the previously reported enrichment of CpG in MeCP2 peaks, we found that the GC% is the predominant predictive feature and that the correlation between MeCP2 binding and CpG% is confounded by the correlation between GC% and CpG%. Integrative analysis further showed that GC% is the dominant feature associated with MeCP2 binding even after including methylation information. This observation was further supported by reanalysis of published mouse brain MeCP2 ChIP-seq data^4^. We found that the GC% continued to be an essential feature in mouse ES cells, but the mCpG% also played an important role in this cell type^17^. Interestingly, while knocking out the methyltransferases *Dnmt1*, *Dnmt3a* and *Dnmt3b* in ES cells modified the MeCP2-binding pattern, it did not significantly change the overall affinity of MeCP2 for chromatin^17^. Thus, the observed difference in binding pattern between olfactory epithelia and ES cells may be due to the large increase in MeCP2 expression throughout neuronal differentiation and a consequent change in the balance between methylation-dependent and methylation-independent binding modes tuned by MeCP2 concentration.

Because numerous genomic features correlate with GC%, we investigated whether the high level of MeCP2 binding in GC-rich regions reflects direct sequence specificity or is confounded by some secondary GC-associated effect. First, previous studies have observed moderate technical GC biases in sequencing library preparation^42^,^43^. To test for such potential biases, we preformed ChIP-qPCR and found good agreement with ChIP-seq. The degree of MeCP2 ChIP enrichment compared with Input is also much stronger than the level of previously reported GC biases. Second, the disappearance of the correlation between differential gene expression and GC% after conditioning on MeCP2 binding (but not vice versa) suggested that it is the local MeCP2 binding that can predict differential expression after MeCP2 KO. Third, *in vitro* binding assays found increased binding affinity for high-GC DNA compared with low-GC DNA, supporting the role of GC% in recruiting MeCP2. Finally, the co-localization of MeCP2 peaks with MNase-seq peaks implied the importance of local chromatin structure in recruiting MeCP2 *in vivo* and suggested that the sequence specificity of MeCP2 might in part arise from its interaction with nucleosomes that themselves prefer GC-rich sequences.

The combined results of our study thus provide support for two binding modes of MeCP2: (1) binding to abundant GC-rich sequences, many of which are found in nucleosomes and (2) binding to mCpG. These results clarify the role of DNA sequence in recruiting MeCP2 and provide functional insights into this important epigenetic regulator.

## Methods

### Animals

Animal care and experimental procedures were approved by the Institutional Animal Care and Use Committee at University of California, Davis (UCD), and were in compliance with the National Institute of Health (NIH) policy. All experiments were performed at UCD. To ensure minimum and equal olfactory stimulation, experimental animals were individually housed in circulating clean air cages for minimum of 24 h before use. Male *Mecp2*^*-/y*^ (KO) and *Mecp2*^*+/y*^ (WT) littermates were obtained by crossing heterozygous *Mecp2*^*-/+*^ female (Jackson Laboratory strain: B6.129P2(C)-Mecp2tm1.1Bird/J, stock number 003890)^44^ with inbred C57BL/6J male.

### Chromatin immunoprecipitation and sequencing

ChIP-seq was performed based on established protocols^18^. Three pilot ChIP assays were performed using two biologically independent samples, each sample consisting of two 8-week-old male mice (C57BL/6J). Each sample was independently ChIPed, and another independent ChIP was later performed on the material remaining from the second sample. Sequencing was performed separately on the three ChIPed DNA libraries, comprising the three pilot ChIP-seq data sets. To obtain deep-sequencing data, independent ChIP assays were performed again as biological replicates. ChIP-seq analyses presented in the paper were largely based on these latter deep-sequencing data sets. For each ChIP assay, two 8-week-old male mice (C57BL/6J) were used to obtain main olfactory epithelium (MOE). Dissociation of MOE was done via trituration in phosphate buffer saline (PBS) with protease inhibitors (Roche, #1183615300). Final number of cells from each experiment was counted and evaluated. Within this preparation, 77.2% of the cells were olfactory sensory neurons, 14% were sustentacular cells and 8.8% were cells from the stroma. MAGnify chromatin immunoprecipitation kit (Life Technologies, Grand Island, NY, USA) was used for all ChIP assays. For the pilot ChIP-seq experiments, 5 × 10^7^ cells and 5 μg of MeCP2 antibody (Diagenode, pAb-052-050) were used. Antibody specificity was confirmed using western blot (Supplementary Fig. 1n). For the deep-sequencing ChIP-seq experiments, 2.5 × 10^8^ cells and 50 μg of MeCP2 antibody were used. ChIP assay was scaled up accordingly (see details below). Protein–DNA complexes were crosslinked with 1% formaldehyde for 5 min at room temperature. Crosslinking was quenched by adding Glycine to final concentration of 0.125 M. After media PBS washing step, MOE cells were incubated in lysis buffer (5 mM PIPES, 85 mM KCl, 0.5% IGEPAL CA-630) on ice for 10 min. We performed gentle dounce homogenization with 10 strokes to release the nuclei and centrifugation to remove supernatant. After adding shearing buffer (50 mM Tris, 10 mM EDTA, 0.1% SDS, 0.5% sodium deoxycholate, pH 8.0) containing protease inhibitors, samples were sonicated for 30 min with 30 s intervals to shear genomic DNA using a Bioruptor 300 (Diagenode, Denville, NJ, USA). Sheared DNA was evaluated by 2100 Bioanalyzer (Agilent Technologies, Santa Clara, CA, USA) to confirm that the fragment sizes ranged between 200 and 300 bp. From the same sample, Input samples were taken and set aside. The remaining sheared genomic DNA preparation was split into two equal portions and incubated with MeCP2 antibody (Diagenode, pAb-052-050) or Rabbit IgG (Millipore, Cat# 12-370) for negative control (IgG ChIPed DNA was examined by qPCR and subsequently was not followed by library construction). ChIP assays were done according to the instructions from the MAGnify ChIP systems. For pilot ChIP-seq libraries, 5 μg of MeCP2 antibody was coupled to 15 μl of Protein A/G Dynabeads for 2 h at 4 °C. For making deep-sequencing libraries, 50 μg of MeCP2 antibody was coupled to 200 μl of Protein A/G Dynabeads for chromatin binding. After adding chromatin to antibody-coupled beads, the tubes were rotated end-over-end at 4 °C for 2 h. MeCP2–genomic DNA complexes bound on the beads were subsequently washed by IP Buffer and prepared for reverse crosslinking. Input DNA samples were included at this step and reversed crosslinked in parallel with the ChIPed samples. Reverse crosslinking was done by DNase-free Proteinase K. MeCP2 ChIP-seq and Input DNA libraries were prepared according to manufacturer's instruction (Bioo Scientific, Austin, TX, USA) using 5143-01 NEXTflex ChIP-Seq kit and 514120 NEXTflex ChIP-Seq Barcodes-6. ChIP and Input DNA were PCR amplified (14 cycles), cleaned up and sequenced on Illumina Hi-Seq 2000.

### Micrococcal nuclease digestion and sequencing (MNase-seq)

For MNase digestion, olfactory neuroepithelia were harvested from *Mecp2* WT littermate adult mice and resuspended in PIPES buffer (5 mM PIPES, 85 mM KCl, 0.5% NP-40, pH 8.0) at 4 °C. After disruption with a dounce homogenizer, nuclei were collected by centrifugation. Collected nuclei were washed once, resuspended in the MNase buffer and digested with 0.5 units of MNase (New England Biolabs, Ipswich, MA, USA) per microlitre volume for 15 min at 37 °C. MNase digestion was stopped by putting the samples on ice and adding EDTA to a concentration of 10 mM. After digestion with 0.1 μg μl^−1^ RNase A (Fermentas, Pittsburgh, PA, USA), DNAs were purified with DNA Purification Magnetic Beads (Life Technologies, Grand Island, NY, USA), and pellets were dissolved in H_2_O. DNA fragments corresponding to mononucleosomes (about 150 bp) were confirmed by using 2100 Bioanalyzer (Agilent Technologies Santa Clara, CA, USA). MNase-seq libraries were prepared as described (Bioo Scientific, Austin, TX, USA) using 5140-01 NEXTflex DNA Sequencing kit and 514101 NEXTflex DNA Barcodes-6. High throughput sequencing was done with Illumina Hi-Seq 2000.

### RNA sequencing

Olfactory neuroepithelia were collected from three different WT and KO mice respectively. Total RNA was extracted with TRIzol Reagent (Life Technologies, Grand Island, NY, USA). The quality of the RNA was determined with an Agilent 2100 BioAnalyzer. Equal amounts of total RNA (13 μg) were used for subsequent isolation of mRNA using Dynabeads mRNA purification kit (Invitrogen). Construction of RNA-seq libraries was done using NEXTflex Directional RNA-Seq kit (Bioo Scientific). cDNA library sequencing was performed with Illumina Hi-Seq 2500 at QB3 Vincent J. Coates Genomics Sequencing Laboratory in the UC Berkeley.

### Bisulfite sequencing

Five-hundred nanograms of DNA was isolated from two wild-type olfactory epithelia (OE) and bisulfite converted using the EZ DNA methylation-Lightning kit (Zymo Research, Irvine, CA, USA). One-hundred nanograms of bisulfite-converted DNA from each OE was then used to make indexed, Illumina sequencing compatible libraries using the Epignome Methyl-Seq Kit (Epicentre, Madison, WI, USA) and Epignome Index PCR Primers (Epicentre). Each bisulfite-converted OE sample had a separate sequencing index allowing for paired end sequencing of both samples in one lane. Whole-genome bisulfite-treated DNA libraries were sequenced with 100 bp paired end reads by Beijing Research Institute (BGI), Sacramento, CA, USA. We performed sequencing in two biological replicates obtaining 173 and 161 million pair-end reads, respectively. Deduplication of the raw sequencing reads resulted in 161 and 152 million reads constituting combined 21 × genomic coverage (computed as the number of sequenced base pairs divided by the genome size). We aligned the deduplicated reads to the mm9 reference assembly using Bismark^45^ package with Bowtie2 aligner, using default Bowtie2 alignment scoring. Methylation calls were performed by Bismark package. The bisulfite conversion rate, estimated using reads mapped to unmethylated mitochondrial DNA, was 99.2%. The methylation level of each CpG in the genome was then estimated as the ratio *l*(*x*)=*n*_m_(*x*)/*n*_tot_(*x*) where *n*_m_(*x*) is the number of reads supporting methylation at position *x* and *n*_tot_(*x*) is the total number of covering reads. After binning the genome we estimated the methylation level *l*(*i*) of each bin *i* as the weighted average of *l*(*x*) using the weights *w*(*x*)=*n*_tot_(*x*). This is equivalent to 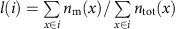 and reduces the variance of the estimate by down weighting CpGs with low coverage. We then estimated the density of methylated, mCpG%, to be *f*_mCpG_(*i*)=*l*(*i*)*f*_CpG_(*i*), where *f*_CpG_ is the fraction of CpG in the window. Bins with *f*_CpG_(*i*)=0, for which *l*(*i*) is undefined, were defined to have *f*_mCpG_=0, but bins with *f*_CpG_(*i*)>0 but all *n*_tot_(*x*)=0 were left undefined. The same method was used for methylated CpH dinucleotides. The methylation levels in murine frontal cortex (GSM1173783)^24^ and embryonic stem cells (GSE30202)^25^ were calculated using the same statistics.

### MeCP2 ChIP-seq and MNase-seq data analysis

The MeCP2 ChIP-seq and Input reads were aligned to the mm9 reference genome using Bowtie 2 (ref. 46). Reads mapping to multiple sites in the genome were discarded. PCR duplicates were removed by keeping at most one mapped read at each position in the genome. The biological replicates were then combined. This yielded 122.1 and 103.7 million reads in the MeCP2 ChIP and Input sets respectively. The fragment coverage values of the ChIP and Input sets were calculated after extending the map coordinate by 200 bp (the experimentally determined fragment length). Published MeCP2 ChIP and Input reads from murine whole brain (GSM494291) (ref. 4), forebrain (GSM1464563, GSM1464564) (ref. 9), hypothalamus (GSM1633577, GSM1633578) (ref. 27), and embryonic stem cells (GSM972976, GSM972981, GSM972995 and GSM1161419) (ref. 17) were processed using the same pipeline. Peaks were called using MACS2 with the narrow peak setting^21^. The MNase-seq reads were also aligned using Bowtie2, but they were not deduplicated. This yielded 101.2 million reads. The nucleosome occupancy was calculated by extending the map coordinates 146 bp and tabulating the coverage.

### Masking

We used two criteria to identify anomalous genomic regions to be masked from downstream analysis. First, plotting the number of genome-wide occurrences (*y* axis) of the nucleosome occupancies (*x* axis) on a log–log scale revealed two distinct types of genomic regions; while the bulk of the genome had moderate coverage, a small fraction of genome—about 0.005%—had anomalously high coverage and highly repetitive sequences. The latter was removed by masking loci with occupancy >100 and the surrounding ±10 kb region. Second, extended regions of low mappability were identified by first calculating the mean of the ENCODE 50 bp mappability track in 1 kb bins and then flagging regions where ten or more consecutive bins have mappability below 50%^47^. These flagged regions and the surrounding ±10 kb were masked.

### Normalization of MeCP2 ChIP-seq enrichment

The relative normalization of the MeCP2 ChIP and Input sets was determined using signal extraction scaling^19^, a method that equalizes the non-enriched background in two sets. Briefly, to separate the ChIP-enriched signal from the non-specific background, we first calculated the list of pairs (*n*_ChIP_, *n*_Input_), where *n*_ChIP_ and *n*_Input_ are the ChIP and Input coverage, respectively, across the genome, and the pairs were ordered by increasing *n*_ChIP_. The cumulative fractions of *n*_ChIP_ and *n*_Input_ along this list of ordered pairs, denoted *f*_ChIP_ and *f*_Input_, respectively, were then plotted against percentile rank in the list (Supplementary Fig. 1d). The ratio *f*_Input_/*f*_ChIP_ evaluated at the percentile of maximal |*f*_ChIP_−*f*_Input_| is the rescaling factor required to match the background contribution to the ChIP-track to that in the Input track, and we found this factor to be *υ*=4.41. To regularize the MeCP2 enrichment in regions with small Input, a small pseudocount *ɛ* was added to the tracks, giving the final enrichment measure 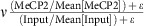. Throughout the paper we use *ɛ*=0.1, but the results are not sensitive to this parameter.

### Signal alignment analysis

To show the genomic landscape around genomic features, we created alignment plots that display the value of a signal track in ±1 or ±4 kb regions surrounding ‘alignment points' as coloured rows in a heat map (Fig. 2). Each alignment point has an associated score (such as the expression level of a TSS or the length of CpG-island) that was used to order the regions (rows), and we indicated this score by using dashed curves. Because of the large number of alignment points, the maps were pixelated to 200-by-200 pixels, and each horizontal line of pixels is thus the average of multiple alignment features. The alignment points we used were TSSs, transcription termination sites, intron/exon boundaries, CpG-islands—all downloaded from the UCSC table browser^48^—and the MeCP2 enrichment-peak centres. The signal tracks were the ChIP-seq and Input coverage, the MNase-seq nucleosome occupancy, GC% and CpG% in tiled 50 bp windows, and the ENCDOE 50 bp mappability track. The former three were normalized by their respective genome-wide mean. Plots of MeCP2 enrichment display the ratio of the pixelated MeCP2 ChIP and Input maps, normalized using signal extraction scaling. For a more quantitative display of the data, we also grouped the alignment points into quartiles of the ordering score and plotted the median signal profile for each quartile.

### GC and CpG dependence of ChIP- and MNase-seq signals

To clarify how the MeCP2 ChIP enrichment and MNase-seq coverage depends on GC% and CpG%, we first tiled the genome with 150 bp windows (chosen to be roughly the size of ChIP-seq and MNase-seq fragments) and recorded the fraction *f*_GC_ of G or C nucleotides and *f*_CpG_ of CpG dinucleotides in each window. We also recorded the values of the three signal tracks—the MeCP2 ChIP-seq and Input coverage and the WT nucleosome occupancy—at the centre of the windows, all normalized by their respective genome-wide mean. We then grouped the windows by the pair (*f*_GC_, *f*_CpG_) and calculated the average of each signal within each (*f*_GC_, *f*_CpG_) group. The MeCP2/Input enrichment was then calculated using these grouped averages. These values were then visualized as a heat maps by linearly interpolating the signals between the observed (*f*_GC_, *f*_CpG_) values. To visualize how the genome-wide correlation between *f*_GC_ and *f*_CpG_ confounds the causal relationship between sequence and signal, we also displayed the contours of the empirical measure *p*(*f*_GC_, *f*_CpG_), each contour labelled by the enclosed genomic fraction. Because CpG is much rarer than CpC, GpC and GpG, the region 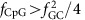 was under sampled and we masked it in the plot.

### Bivariate distributions

We created the bivariate distribution plots of the ChIP-seq coverage and nucleosome occupancies (Supplementary Fig. 1a) by first tabulating the number of times the two signals *s*_1_ and *s*_2_ are observed as a pair (*s*_1_, *s*_2_) and then normalizing these counts to get an empirical density function. The contours of this density function were then created by first interpolating between the observed points (*s*_1_, *s*_2_) and then placing contours evenly between zero and twice the mean density in the plotted region. To create the bivariate distributions of the ChIP-seq and MNase-seq signals versus GC% and CpG%, we first tabulated both the nucleotide content in tiled 150 bp windows and the value of the signal track in the centre of these windows and then proceeded as above. The MeCP2 ChIP, Input and MNase-seq signals were all normalized by their respective genome-wide mean.

### Random Forest regression for MeCP2 binding

To improve our understanding of how sequence determines MeCP2 binding of DNA, we built a machine learning programme that predicts MeCP2 binding based on the concentration of k-mers in the bound sequence. This programme puts a lower bound on the degree to which one can predict the level of MeCP2 binding only from local concentration of k-mers in DNA sequence and also determines the relative importance of various k-mer concentration in the DNA sequence on MeCP2 binding.

We used Random Forest regressor, a non-parametric supervised learning method used for classification and regression that is particularly suitable for our goal, as it can capture the potentially complex relationships between sequence features and MeCP2 binding. We divided the mouse genome into 200 bp windows and used mono-, di- and trinucleotide concentrations (removing features that are equivalent under reverse complement symmetry) as predictors of the window averaged fold enrichment obtained by MACS2 peak caller from MeCP2 ChIP-seq signal and Input. We used 10,000 randomly sampled 200 bp windows across the uniquely mappable regions of mouse genome as a training set for a 100 tree Random Forest regressor. Our programme uses scikit-learn implementation of Random Forest regressor with CART algorithm.

We first trained the Random Forest regressor on mono-nucleotides only. Since the concentration of all mono-nucleotides adds up to 1 and the concentration of C equals that of G due to the strand symmetry and similarly for A and T, the only independent feature is the GC%. The Pearson correlation between the true MeCP2 ChIP-seq enrichment and that predicted based on GC% was 84% across the mappable part of genome. We further used the regressor to classify sites as MeCP2 bound or unbound by thresholding of regressor predicted value. We compared this classification with prediction of MeCP2 binding by MACS2 peak caller, where we required that a predicted MeCP2-bound window has at least 50 bp overlap with MACS2 peak to be called a true positive. ROC of this classifier in shown in Fig. 1b, and the AUC of ROC is 94%. We investigated robustness of our results with respect to varying depth of regression trees and found that AUC varies <1%, and correlation between prediction of MeCP2 enrichment and its observed values varies <2% as the depth of regression trees varies between 3 and 8.

Next, we added the dinucleotide concentrations into the feature set. Since we now have multiple features in the set, we are interested in how important the individual features are for the prediction of MeCP2 enrichment. The average relative tree-depth of a feature used in trees of the forest is used to quantify the relative importance of the features on the predicted value. Our Random Forest regressor showed that dinucleotides have very small importance and hence a negligible impact on the prediction of MeCP2 binding. GC% has importance of 88%, while the second most important feature, the GpC dinucleotide has importance of only 2.3%. Furthermore, the importance of CpG falls consistently below the importance of CpC/GpG and GpC. Relative importance of features is shown in Fig. 1c. Due to the negligible importance of dinucleotides, the AUC of the ROC and the Person correlation showed little improvement with inclusion of dinucleotides; AUC only improved by 0.5% and Pearson correlation by 1.2%. Similarly, inclusion of both di- and tri- nucleotides showed negligible importance of all di- and tri- nucleic features and little improvement in ROC and Pearson correlation. With both di- and tri- nucleotides included in the feature set, the importance of GC% is 87%, the highest di-nucleotide GpC has importance of 2.3% and the highest trinucleotide CAG has importance of 0.5%. Similar results were obtained as depths of regressors ranged between 3 and 8.

### Expression analysis

The RNA-seq reads were aligned to the mm9 refGene using TopHat 2 (refs 49, 50). Only reads mapping uniquely, in proper pairs, and to the same chromosome were retained, leaving 65, 55 and 56 million reads in the WT replicates and 58, 51 and 51 million reads in the KO replicates. Differential gene expression and statistical significance were calculated for the refGene transcripts using cufflinks and cuffdiff^51^, ran with default parameters but keeping only uniquely mapped and proper read pairs.

### Gaussian graphical models

In a Gaussian graphical model the weight of the edge between two random variables *X*_*i*_ and *X*_*j*_ is the full partial correlation 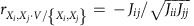 where *J*_*ij*_ is the inverse of the correlation matrix *r*_*Xi*,*Xj*_. The pairwise correlation all pairs of random variables were calculated after removing filtered regions and, when relevant, regions with undefined methylation density.

### Differential expression aggregated by gene annotations

We identified significantly downregulated gene ontology terms by first calculating the aggregated fold change for each gene ontology (GO) term—defined as the mean of the expression change log_2_WT/KO across all associated genes—and then ranking the GO terms by this aggregated fold change. We used the GO Consortium's mouse annotation (gene_association.mgi and go-basic.obo downloaded on 13 March 2015) and restricted the analysis to genes with expression larger than 1 FPKM (geometric mean between WT and KO) and to GO terms with 10 or more such genes^52^. The FPKM values were pooled for genes with multiple refSeq transcripts. To assess the significance of the expression changes, we first quantified the dispersion of the differential expression by binning the genes by 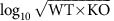, calculated the mean and variance of the fold changes within each bin, and performed quadratic fits for the fold and variance. Using these dispersion fits we then calculated the *Z*-score of the differential expression for each gene and calculated the significance of the aggregated differential expression using the *t*-test. We finally used the Benjamini–Hochberg procedure to control the false discovery rate.

## Additional information

**Accession codes:** All sequencing data have been deposited in the Gene Expression Omnibus under the accession code GSE71126.

**How to cite this article:** Rube, H. T. *et al*. Sequence features accurately predict genome-wide MeCP2 binding *in vivo*. *Nat. Commun.* 7:11025 doi: 10.1038/ncomms11025 (2016).

## Supplementary Material

Supplementary Information Supplementary Figures 1-10, Supplementary Tables 1-3, Supplementary Methods and Supplementary References
