# Supplementary material for: Sequence features accurately predict genome-wide MeCP2 binding in vivo
Source: Nat Commun. 2016 Mar 24;7:11025. doi: 10.1038/ncomms11025 (PMC4820824; doi:10.1038/ncomms11025)
Supplement: Supplementary Information — Supplementary Figures 1-10, Supplementary Tables 1-3, Supplementary Methods and Supplementary References [file ncomms11025-s1.pdf]

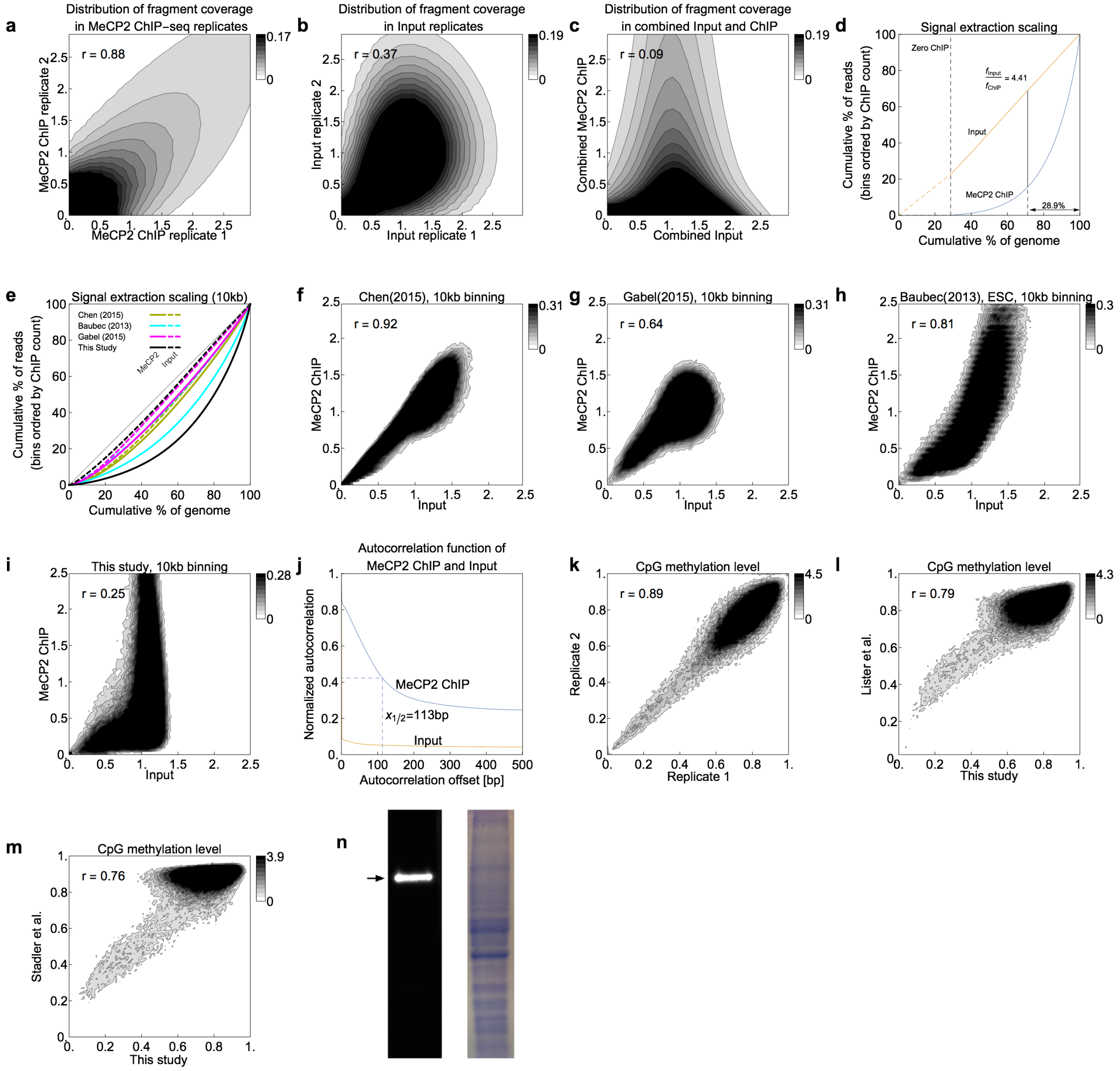


**SUPPLEMENTARY FIGURE 1:** Comparisons of the biological replicates of ChIP-seq, Input and Bisulfite-seq. (**a**) Density plot of MeCP2 ChIP-seq genome coverage (calculated using tiled 150 bp windows) shows high correlation between the replicates. (**b**) Density plot of Input in replicates. (**c**) Density plot of combined MeCP2-ChIP vs. combined Input shows no correlation. (**d**) Signal extraction scaling plot used for inferring the relative normalization of the ChIP and Input tracks. The pairs of read coverage in ChIP and Input ($n_{\mathrm{ChIP}}$, $n_{\mathrm{Input}}$) are calculated across the genome, and this list of pairs is ordered by $n_{\mathrm{ChIP}}$. The curves indicate cumulative fraction (y-axis) of $n_{\mathrm{ChIP}}$- (blue) and $n_{\mathrm{Input}}$-reads (yellow) as a function of rank in the list (x-axis). The black vertical line shows the point of maximal difference between MeCP2 ChIP and Input and indicates the fraction of genome enriched by the ChIP. (**e**) Signal extraction scaling analysis of published MeCP2 ChIP-seq data sets using 10 kb windows[1-3]. (**f-i**) Density plot of MeCP2 ChIP vs. Input for the published data sets and for this study using 10kb binning. The X chromosome was filtered out since male mice only have one copy (and thus half the coverage). (**j**) Autocorrelation function of MeCP2 ChIP and Input using strand-specific counts of mapped reads at single base pair resolution. The correlation functions have been normalized for sequencing depth as described in the Supplementary Methods section. (**k**) Distribution plot of the methylation levels in the two biological replicates using 10 kb binning. (**l**) Same as **k** but comparing this study to Lister et al.[4]. (**m**) Same as **k** but comparing this study to Stadler et al.[5]. (**n**) Full-length Western blot showing the antibody specificity. Main olfactory epithelium (MOE) was harvested from two adult C57BL/6J mice. Cell lysate was obtained by homogenization in 300 μl of Tris Buffered Saline pH7.2 with proteinase inhibitors (Roche, #11836153001) followed by addition of 4X SDS PAGE loading dye. Aliquots (10 μl) of MOE extract were loaded on 10% SDS PAGE and stained with Coomassie blue, shown on the right; an equal amount of the extract was used for western blotting using 1.2 ng $\mu l^{-1}$ of MeCP2 antibody (Diagenode, pAb-052-050), shown in the left. Arrow indicates positive band for MeCP2 binding.

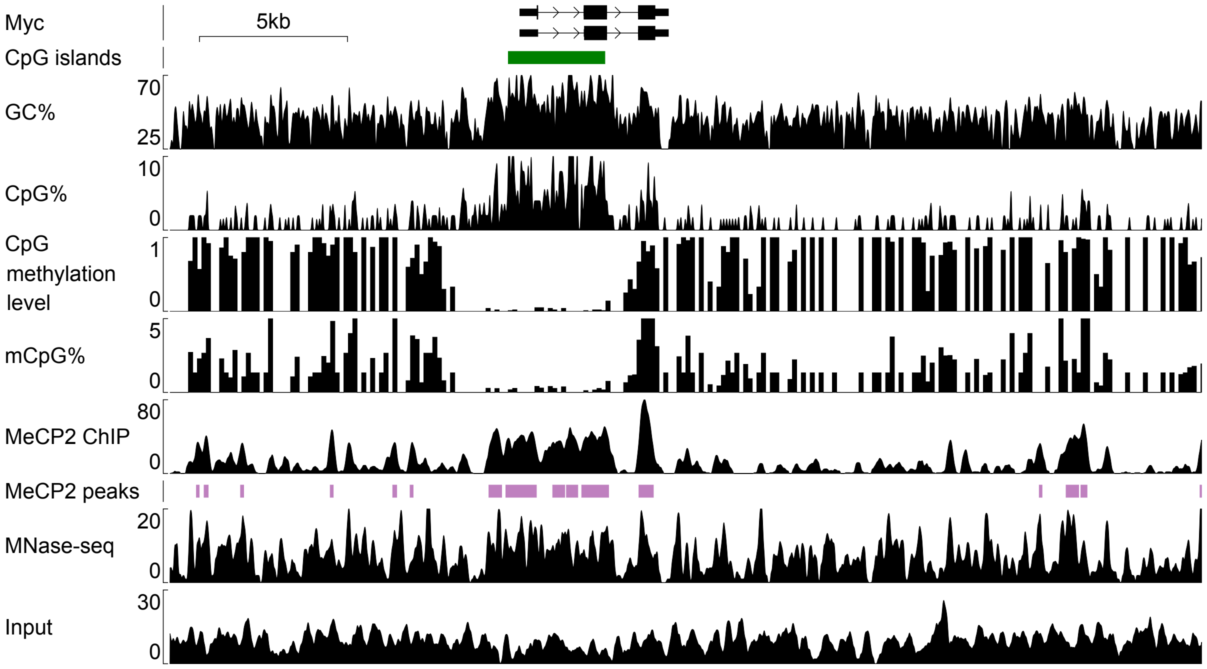


**SUPPLEMENTARY FIGURE 2**: Sequence GC content, CpG methylation, MNase-seq coverage and ChIP-seq coverage in the region surrounding *Myc*.

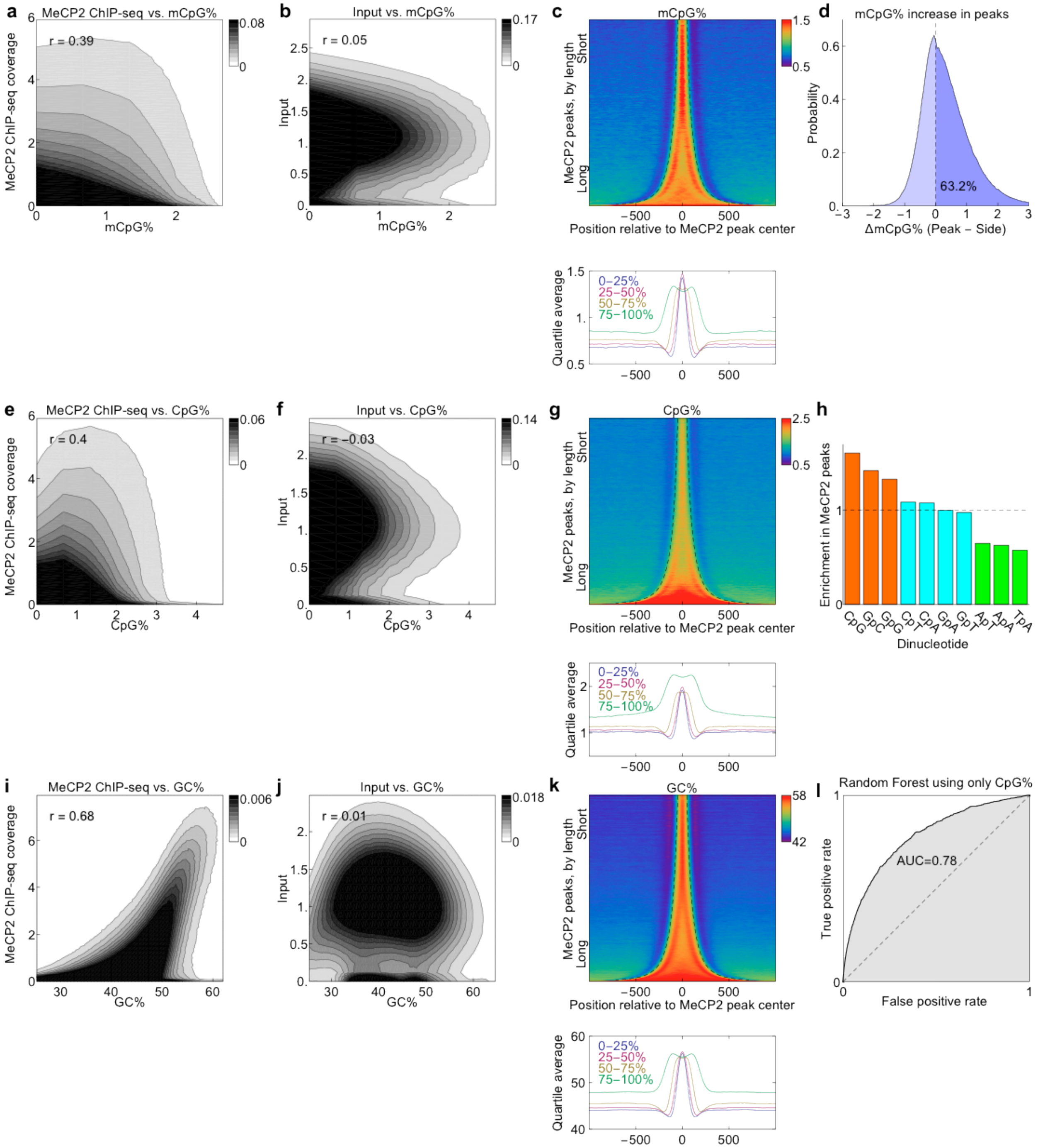


**SUPPLEMENTARY FIGURE 3**: Sequence and methylation content versus ChIP-seq and Input fragment coverage. (**a**) Distribution plot showing the probability density (shade) of mCpG% vs. MeCP2 ChIP-seq fragment coverage. (**b**) Same as **a** but showing mCpG% vs. Input. (**c**) mCpG% (color) in regions surrounding MeCP2 peaks (dashed lines). The bottom plot shows the median CpG% in peaks organized into peak width quartiles. (**d**) Distribution of the difference in mCpG% between peaks and the adjacent regions. (**e**) Same as **a** but showing CpG% vs. MeCP2 ChIP-seq. (**f**) Same as **a** but showing CpG% vs. Input. (**g**) CpG% (color) in regions surrounding MeCP2 peaks. (**h**) Dinucleotide enrichment in MeCP2 peaks relative to peaks shifted randomly by ±5 kb. The dashed line indicates no enrichment. (**i**) Same as **a** but showing GC% vs. MeCP2 ChIP-seq. (**j**) Same as **a** but showing GC% vs. Input. (**k**) GC% (color) in regions surrounding MeCP2 peaks. (**l**) ROC-curve for predicting MeCP2 ChIP-seq peaks using Random Forest regressor based on CpG% in 200 bp windows. Plots **a-c**, **e-g** and **i-k** use 150 bp windows.

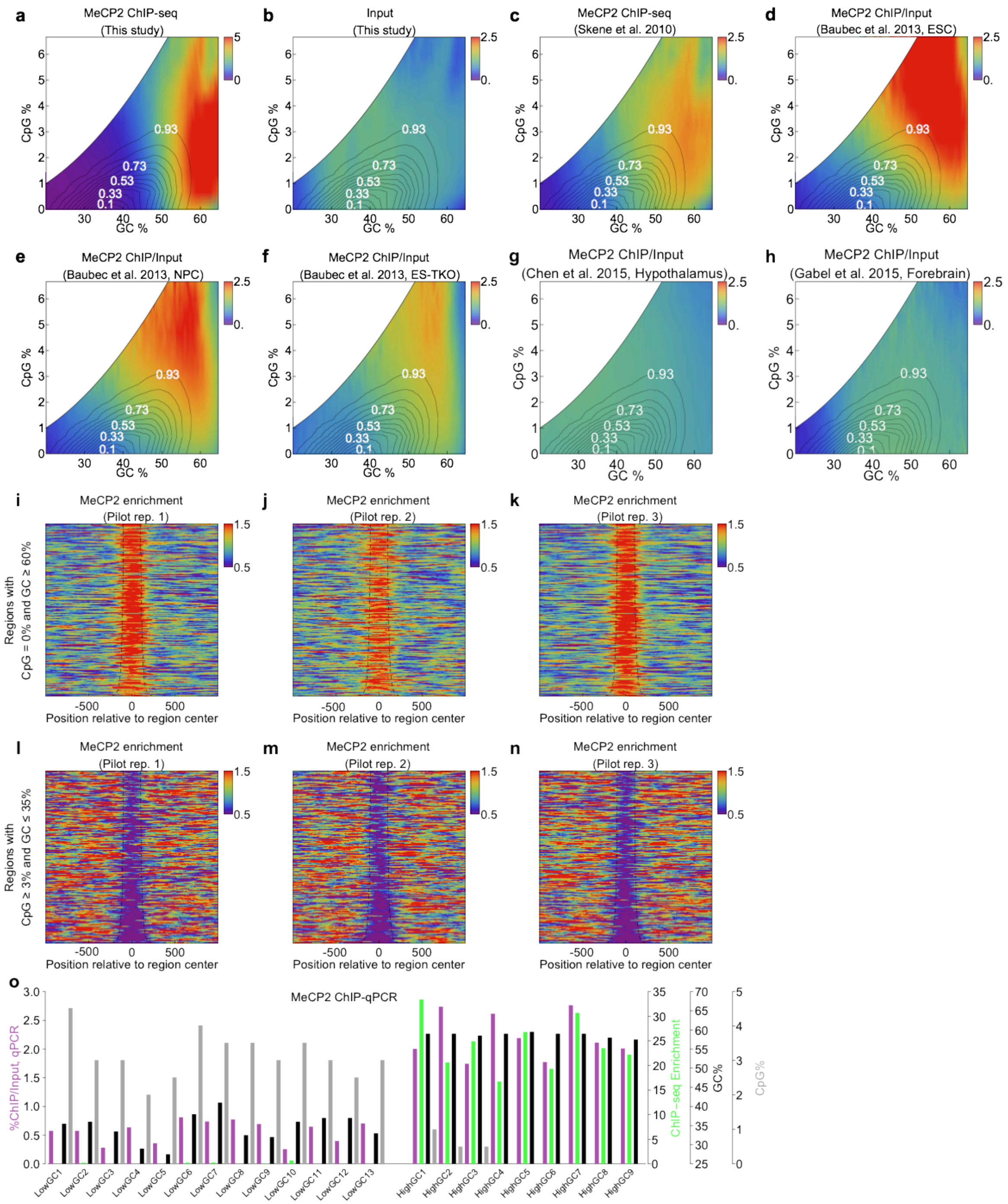


**SUPPLEMENTARY FIGURE 4**: GC%- and CpG%-dependence of (**a**) MeCP2 ChIP-seq, (**b**) Input, (**c**) MeCP2 ChIP-seq from Skene et al. 2010, (**d**) MeCP2/Input in ES cells from Baubec et al.[1], (**e**) MeCP2/Input in ES cell-derived neuronal progenitor cells (NPC) from Baubec et al.[1], (**f**) MeCP2/Input in Dnmt1, Dnmt3a, and Dnmt3b triple knockout (TKO) ES cells from Baubec et al.[1], (**g**) MeCP2/Input in hypothalamus from Chen et al.[3], (**h**) MeCP2/Input in forebrain from Gabel et al.[2]. MeCP2 enrichment was normalized by sequencing depth in **d-h**. (**i-n**) Same as Fig. 2a,e but using three pilot MeCP2 ChIP-seq data sets. (**o**) ChIP-qPCR validation (purple) of MeCP2 ChIP-seq profile (green) in regions with either high GC% and low CpG% or low GC% and high CpG%. Three technical replicate measurements were performed on each of three biological replicate samples. The primer coordinates and sequences are given in Supplementary Table 2. 200 bp windows centered at the qPCR regions were used for calculating GC% and CpG%.

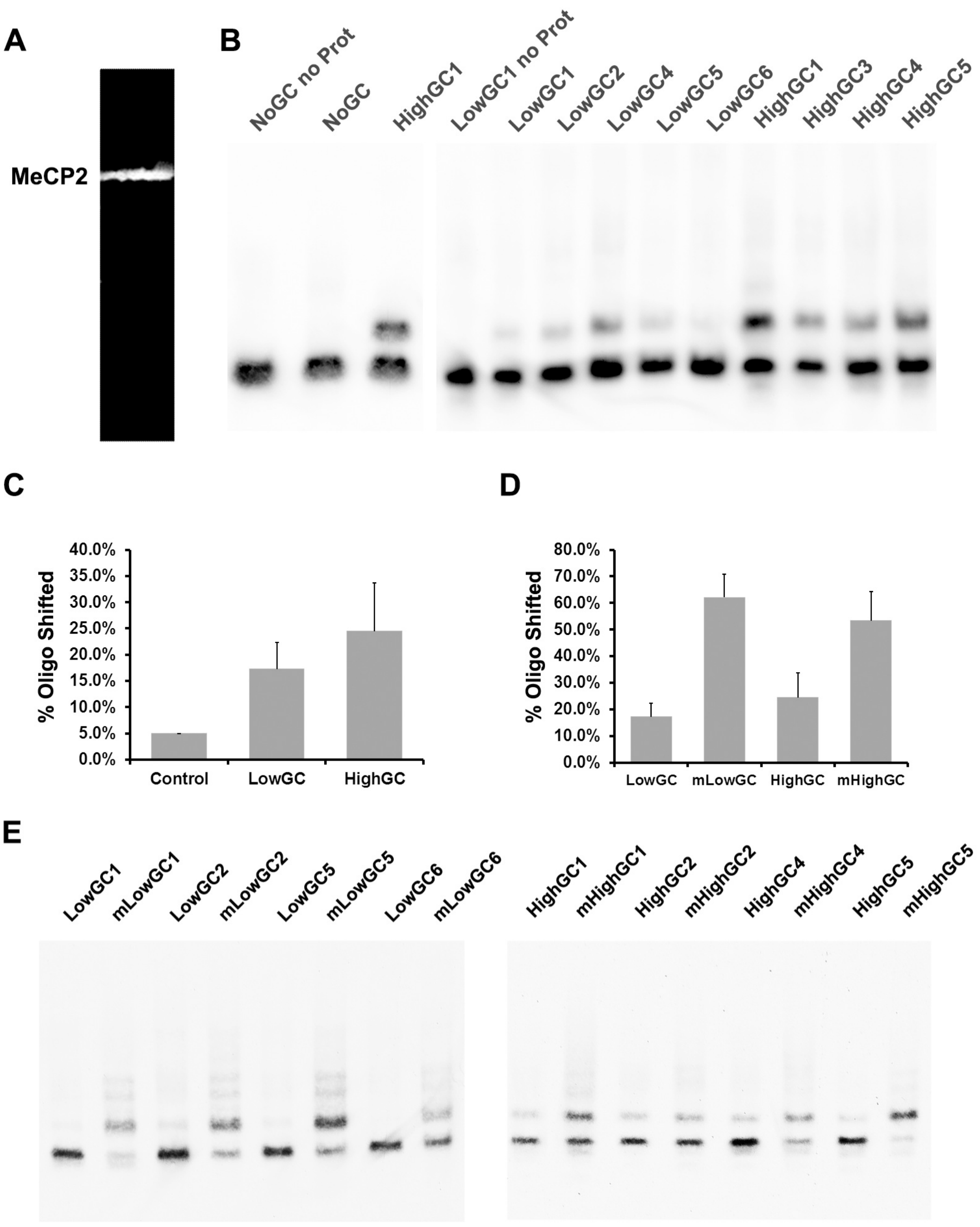


**SUPPLEMENTARY FIGURE 5:** MeCP2 binding affinity evaluated by EMSA. (**a**) Recombinant MeCP2 isoform2 purified from bacterial lysate via FPLC and confirmed by western blotting. (**b**) Equal amounts of MeCP2 were used to evaluate binding to double-stranded unmethylated oligos containing different GC% (see Supplement Table 3 for oligo information; representative MeCP2-oligo bindings are shown). All EMSA reactions contains poly-dA:dT as competitor DNA (see supplemental methods for detail). Serving as control, first two lanes show an oligo with no GC (Biotin-attattattattattattattattattattattattattattattat) in the binding reaction without and with the presence of MeCP2 protein, respectively. In addition, the fourth lane also without the presence of MeCP2 serves as a control to indicate the location of unbound oligos. (**c**) Densitometry analysis of the EMSA blots. With 6 lowGC and 8 highGC oligos tested, higher affinity of MeCP2 towards highGC oligos was observed (mean±SD: 17.3±5.0% vs. 24.5±9.2%; one-sided t-test p-value = 0.04; 4 rounds of EMSA blots were performed for each oligo). (**d**) MeCP2 binds methylated oligos with higher affinity. Each methylated oligo contains the same sequence as the non-methylated version and has one symmetrically methylated CpG on both strands. 2 rounds of EMSA blots were performed for each oligo. Error bars in **c** and **d** indicate standard deviation. (**e**) Representative MeCP2-DNA interactions are shown, comparing side-by-side the MeCP2 binding to methylated and unmethylated oligos.

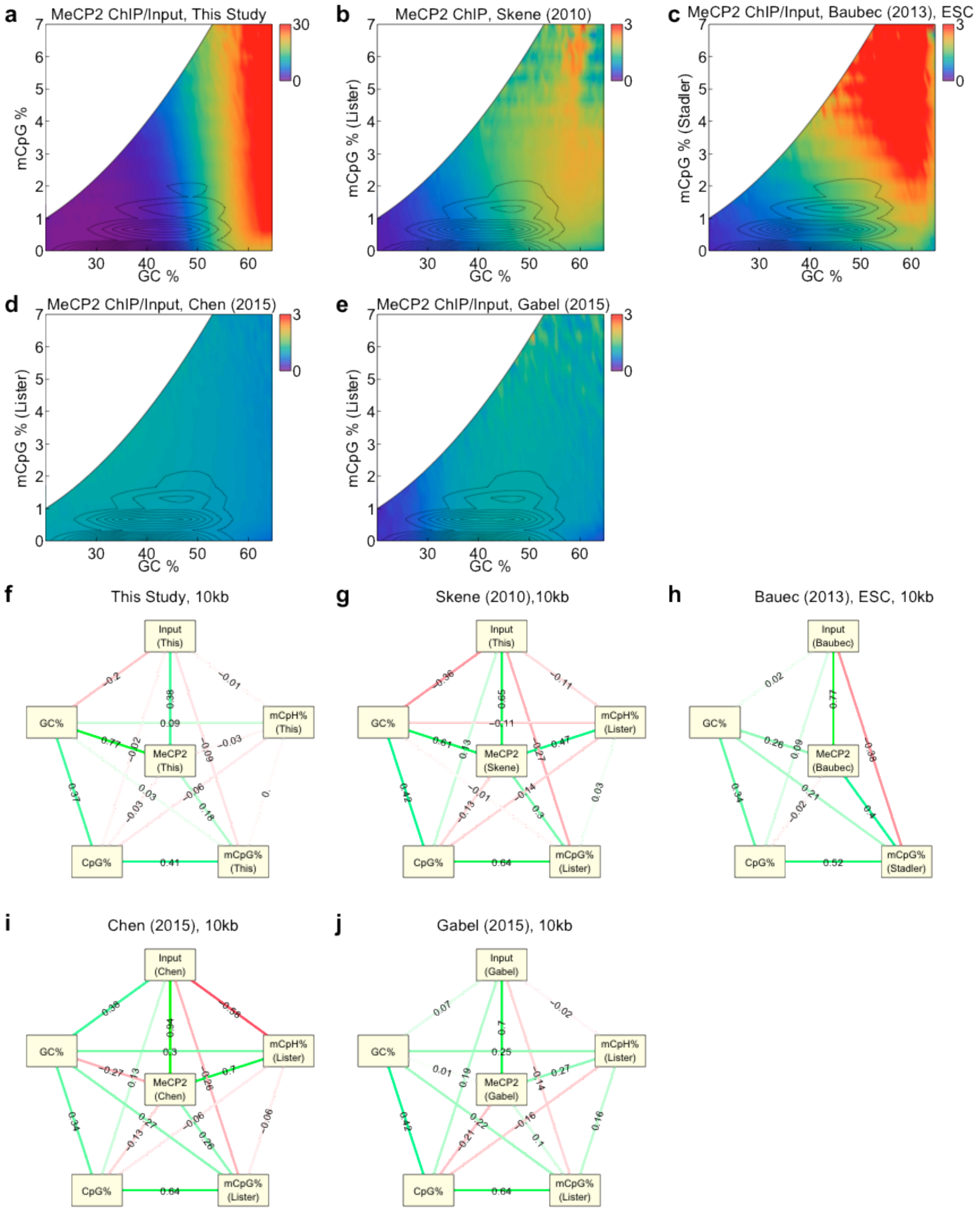


**SUPPLEMENTARY FIGURE 6:** Methylation- and sequence-dependence of MeCP2 ChIP enrichment. (**a**) GC%- and mCpG%-dependence of the mean MeCP2 enrichment (colors) calculated using 150 bp windows and signal extraction scaling. (**b**) Same as **a** but using MeCP2 ChIP-seq data from Skene et al.[6] (no Input was available) and methylation data from Lister et al.[4]. (**c**) Same as **a** but using ESC MeCP2 ChIP-seq and Input data from Baubec et al.[1] and methylation data from Stadler et al.[5]. (**d**) Same as **a** but using MeCP2 ChIP-seq and Input data from Chen et al.[3] and methylation data from Lister et al.[4]. (**e**) Same as **a** but using forebrain MeCP2 ChIP-seq and Input data from Gabel et al.[2] and methylation data from Lister et al.[4]. MeCP2 enrichment was normalized by sequencing depth in **c-e**. (**f-j**) Gaussian graphical models describing the full partial correlations between the MeCP2 ChIP, Input, GC%, CpG%, mCpG% and mCpH% at 10 kb resolution, see Fig. 3b-f and Methods.

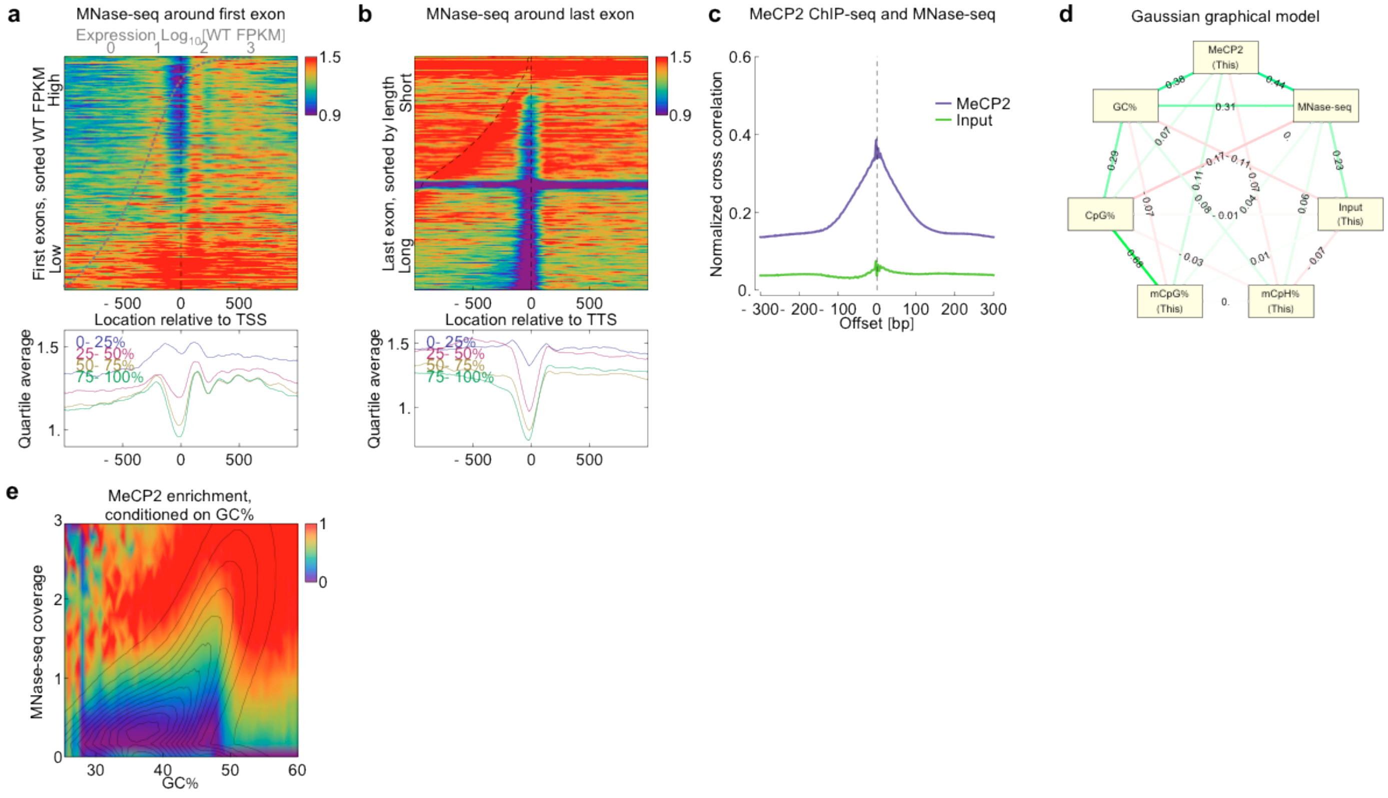


**SUPPLEMENTARY FIGURE 7:** Structure of the MNase-seq profile. (**a-b**) Alignment plots for WT MNase-seq read densities (color) around TSS and TTS. The dashed vertical lines indicate TSS and TTS, respectively. The TSSs are sorted by expression, as indicated by curved gray dashed line. The TTSs are sorted by length of the last exon, and the dashed black curves indicate the outlines of the exons. (**c**) Normalized cross-correlation between MeCP2 (ChIP and Input) and MNase-seq. The correlation coefficients have been normalized for sequencing depth (see Supplementary Methods). (**d**) Gaussian graphical model after including the MNase-seq coverage, see Fig. 3b. (**e**) Dependence of MeCP2 enrichment on nucleosome occupancy. At each value of GC%, the MeCP2 enrichment was linearly transformed to span [0,1] in order to show the conditional dependence of MeCP2 on nucleosome occupancy (color). The contours indicate the genome-wide joint distribution *p*(GC%,MNase-seq).

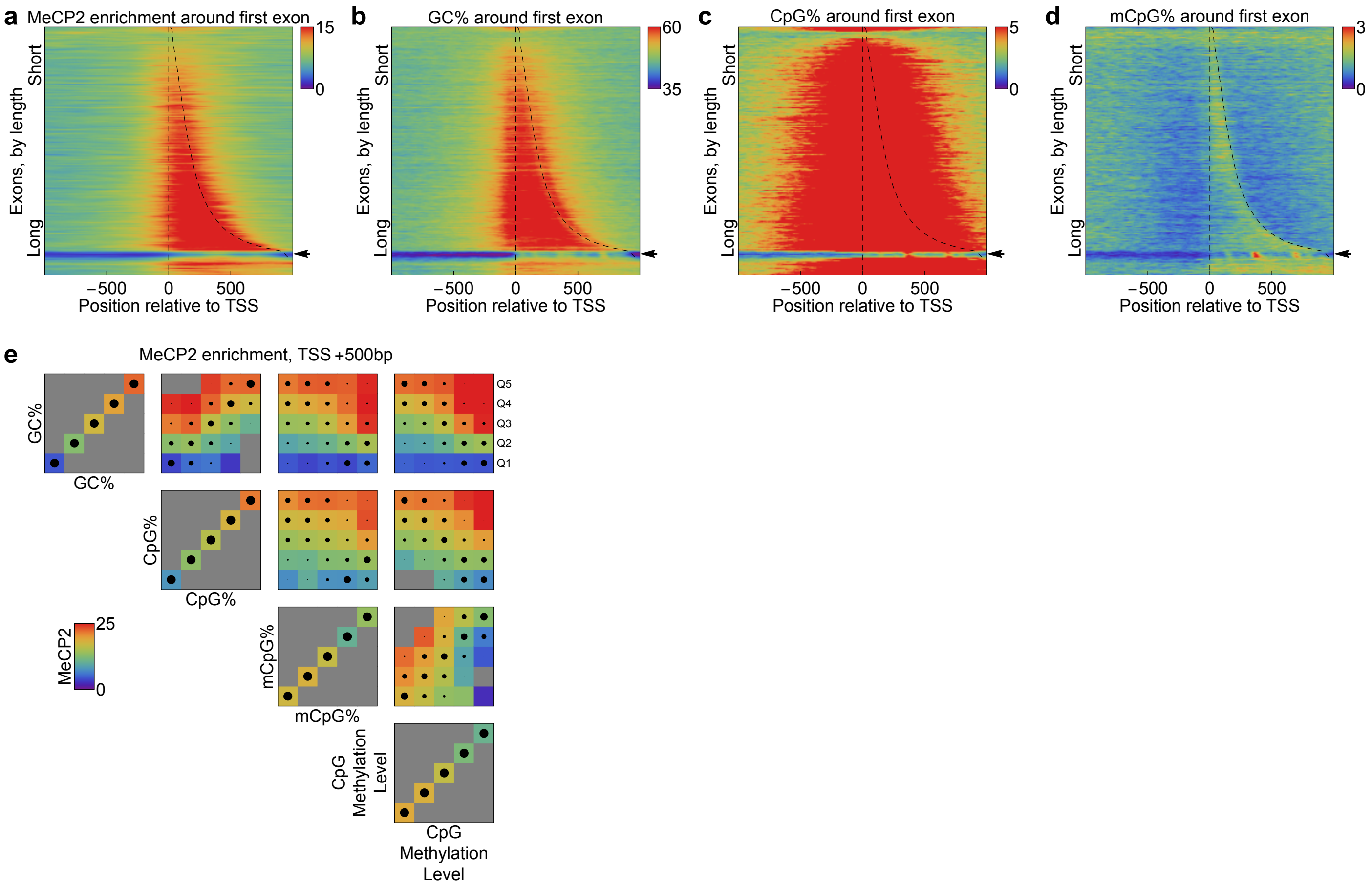


**SUPPLEMENTARY FIGURE 8:** MeCP2 binding and sequence content around TSSs. (**a**) Alignment plots showing MeCP2/Input-enrichment (color) around TTSs. (**b**) Same as **a** but showing aligned GC%. (**c**) Same as **a** but showing aligned CpG%. (**d**) Same as **a** but showing aligned mCpG%. A cluster of genes (arrows) displays low MeCP2 binding, GC% and CpG%. These genes have length in the range 930 bp to 950 bp and 79% of them are Olfr gene family members, which are known to have low GC%[7]. (**e**) GC%, CpG%, mCpG% and CpG methylation level dependence of the MeCP2 enrichment 500 bp downstream of TSSs. Each subplot shows how the mean MeCP2 enrichment (color) depends on a pair of covariates after grouping by quintile (columns and rows in subplots). Gray indicates quintile combinations without data. The areas of the black disks show the conditional probability of a column in a subplot given a row (or vice versa). The direction of the color gradient indicates the strongest dependence of the MeCP2 enrichment. Bands of large disks indicate variables that co-vary; e.g. GC% and mCpG% anti-correlate.

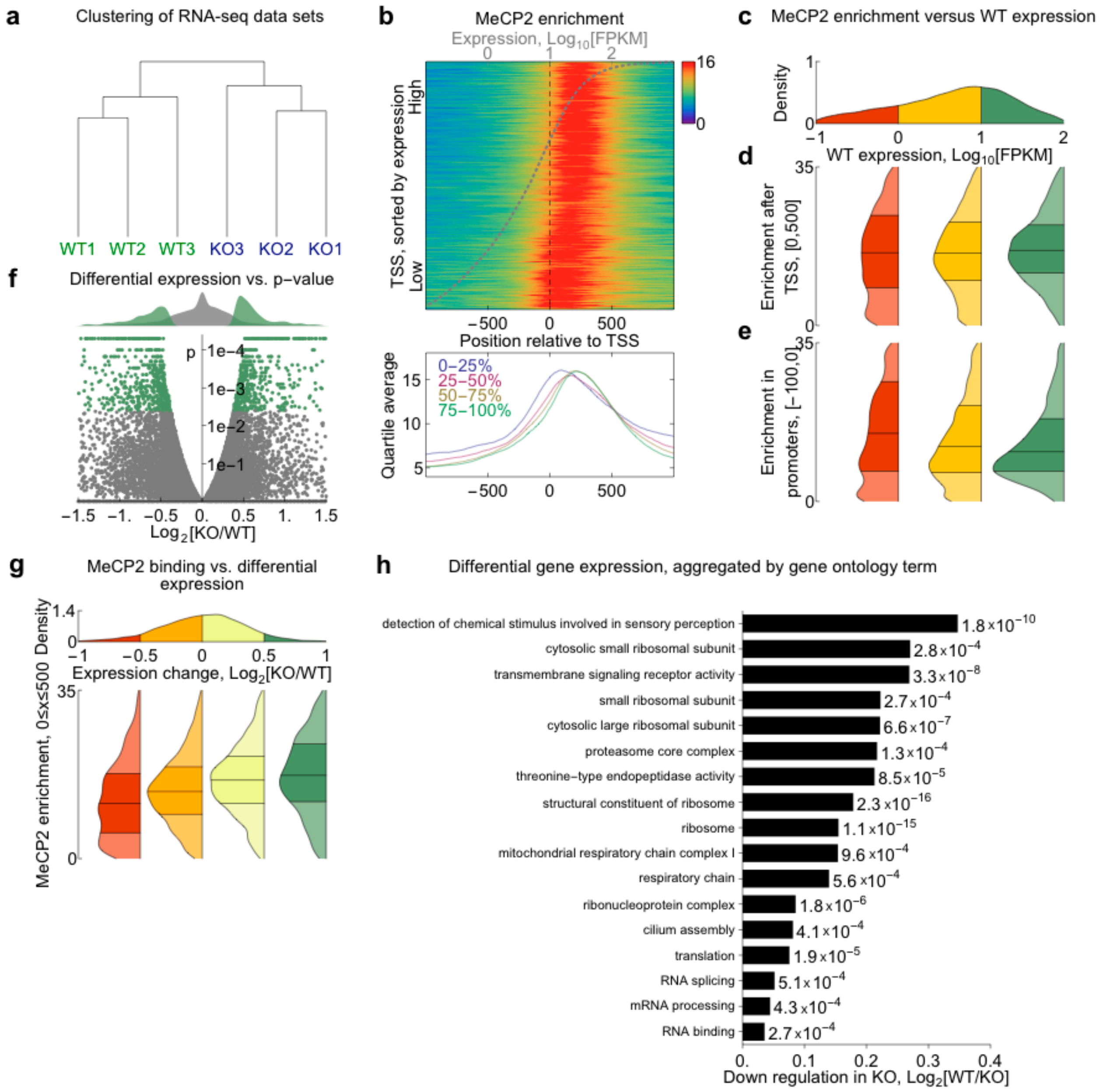


**SUPPLEMENTARY FIGURE 9:** Relationship between MeCP2 enrichment and expression levels. RNA-seq experiments were performed in biological triplicates, each yielding between 51 M and 65 M mapped reads. (**a**) Hierarchical clustering of WT and KO expression values Log[1+FPKM] using Euclidean distance metric and mean linkage function. (**b**) Aligned MeCP2-enrichment profile (color) around TSSs (vertical black dashed line), ordered by expression value (curved gray line). Only transcripts with expression exceeding 0.1 FPKM are shown. The bottom plot shows the median MeCP2 enrichment profile in genes organized by expression quartile. (**c**) Distribution of WT expression levels discretized using equidistant thresholds (colors). (**d**) Distributions of MeCP2 enrichments in the 5' end of genes (defined to cover 500 bps downstream of the TSS) with expression levels thresholded as indicated in **c**. Darker shades indicate the central two quartiles. (**e**) Same as **d** but showing MeCP2 enrichment in promoters (defined to cover 100 bps upstream of TSS). (**f**) Volcano-plot showing fold change KO/WT versus significance. Green dots are significantly differentially expressed transcripts (Benjamini $p<0.05$). (**g**) MeCP2 enrichment versus expression change. The top plot shows the distribution of expression fold changes between KO and WT. The genes are binned using equidistant thresholds (colors); the bottom vertical plots show the distribution of the MeCP2 enrichment 500 bp downstream of corresponding TSSs, with the darker shades indicating the middle two quartiles. (**h**) Gene ontology terms with significantly down-regulated expression in the KO. Bars indicate the mean fold change of the annotated genes, and numbers indicate the Benjamini-Hochberg FDR. Only terms with $q<0.1\%$ are shown.

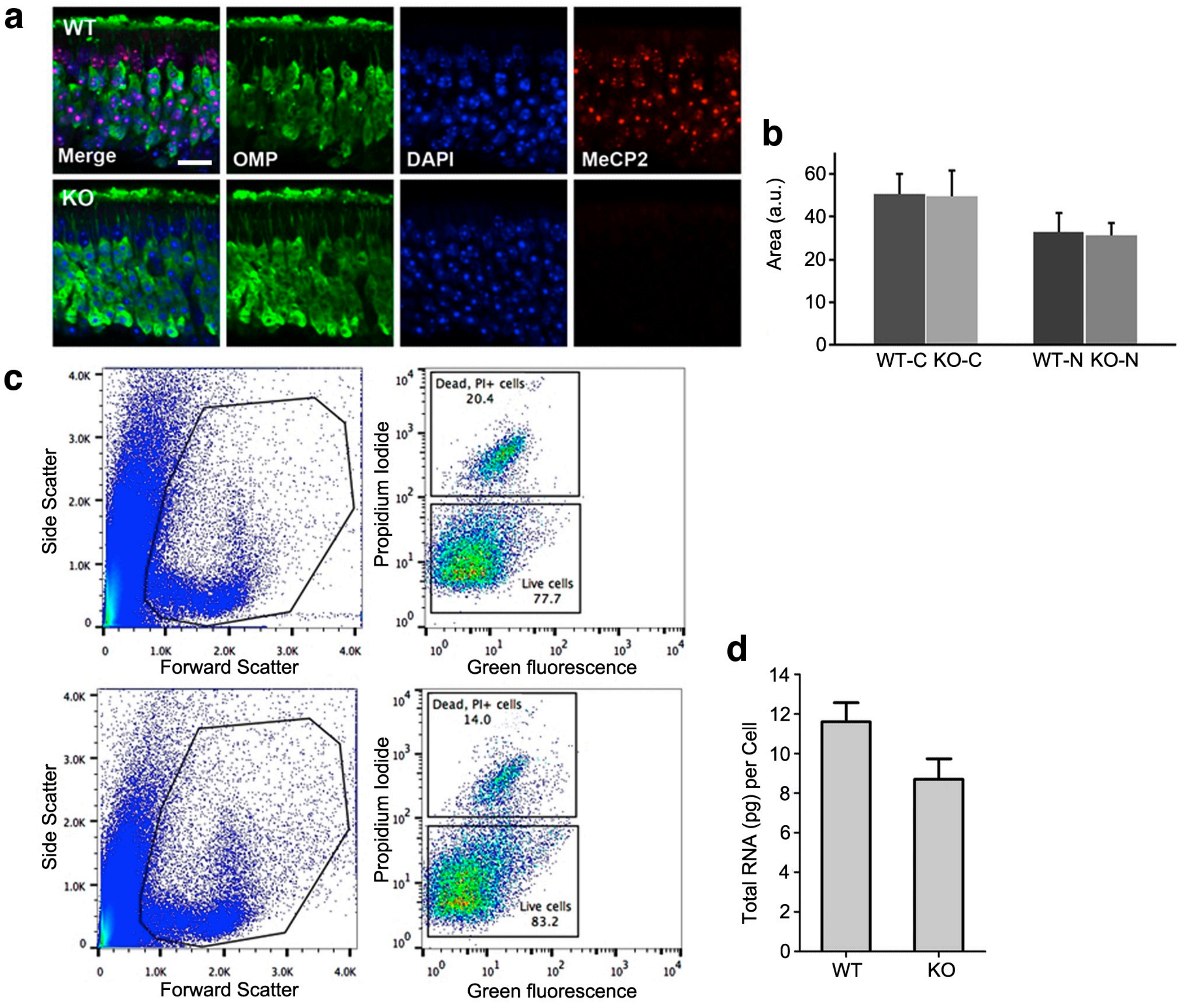


**SUPPLEMENTARY FIGURE 10**: Measurements of changes in total RNA and cell size between wild-type (WT) and *Mecp2* knockout (KO) olfactory epithelia (OE). (**a**) To compare cell body and nucleus size of olfactory sensory neurons in WT and KO, OE were immunostained with antibodies against olfactory marker protein (OMP, 1:1000, green), MeCP2 (1:1000, red) and counterstained with DAPI for nuclei (blue). MeCP2 signal is absent in the KO as expected. Scale bar represents 20 μm. (**b**) Soma and nucleus sizes were measured in the OE and compared between WT and KO. No difference between cell body (WT-C, $n$=32; KO-C, $n$=34, $p > 0.75$) and nuclei (WT-N, $n$=34; KO-N, $n$=34, $p > 0.30$). (**c**) To measure changes in total RNA in the OE, olfactory neuroepithelia were dissociated and viable cells are sorted through FACS. Viability of OE cells was indicated as propidium iodide negative and collected and counted. (**d**) Total RNA from the same numbers of viable OE cells from WT ($n$=2) and KO ($n$=3) were extracted and measured. Decreased total RNA level was observed in KO when compared to that of the WT (11.6 ± 0.97 pg/cell versus 8.75 ± 1.03 pg/cell). Error bars in **b** and **d** indicate standard deviation.

**SUPPLEMENTARY TABLE 1**. Realtime PCR primer information for ChIP-seq validation in the BDNF region.

| | |
|---|---|
| BDNF-1F | AGAATATCAATGCTGGGACTGC |
| BDNF-1R | TCAGCGTACAGTGTGCTTTCTT |
| BDNF-2F | GCATATAAAGTTTGCGTGTCCA |
| BDNF-2R | CCATCCTGAAAAACGTAGAAGG |
| BDNF-3F | TCTGCTATGCTTTCTCTAGTTCCA |
| BDNF-3R | TTGCAGAATGGAAGAGAAGTCA |
| BDNF-4F | CTGACAACCGTCTTGTATTCCA |
| BDNF-4R | TAACCCACTCTTACCCTCTCCA |
| BDNF-5F | CAGGTATTCTTTTCCTCGCTGT |
| BDNF-5R | CGCTCCAAAATCTGACTCTCTC |
| BDNF-6F | GGTGTGTGTGCGTGTACAGTAG |
| BDNF-6R | TCCCTCCATCCTCTTGTTCTAA |
| BDNF-7F | CAATGAAAACATGCTCCAAGAA |
| BDNF-7R | GCTGTTATGGGGTTATCCTGTC |
| BDNF-8F | AGAGGTTCAGACAACCAGATCC |
| BDNF-8R | AAAGTGCCAAATACCTTGGTTG |
| BDNF-9F | GGAGCACATACTGATCTTGCAG |
| BDNF-9R | TGTGCAGGTATCTCTGGAACTG |
| BDNF-10F | GAAATTGATGGGGTGATGATTT |
| BDNF-10R | GGTTATGCAGCTCTCTTGACCT |
| BDNF-11F | GCACCACAATTACTTCATGCTT |
| BDNF-11R | ACTGGGTTTCAGGACATTGAGT |
| BDNF-12F | GTCCAAAGACCACTCTCTGCTC |
| BDNF-12R | GCCAAGCCTGTCATTATCTAGG |
| BDNF-13F | GTGGACTCCCACCCACTTT |
| BDNF-13R | TCCATTTGATCTAGGCAGAGG |
| BDNF-14F | GGTGGGTTTCTTTTCTTTCTCTT |
| BDNF-14R | CCGGATAGCATTACACCAAGTT |
| BDNF-15F | GAAGCGTGACAACAATGTGACT |
| BDNF-15R | CACAACTTCTCAGTCCCCTAGC |
| BDNF-16F | TACGGGTTTGATTCCTCTAAGC |
| BDNF-16R | CGCCTGACTAAGCCTATTGTGT |

**SUPPLEMENTARY TABLE 2.** Realtime PCR primer and amplicon information for ChIP-seq validation of GC%-associated MeCP2 binding.

| Name | Sequence | Amplicon Locus (mm9) |
|---|---|---|
| HighGC1-F | CAGCTAACACAGAGGCCACA | chr1:132,950,969-132,951,062 |
| HighGC1-R | GCTACAGGGACTCCTGCTTG | |
| HighGC2-F | AAGTCTCAGGGTCTCCCCATACA | chr2:163,190,044-163,190,129 |
| HighGC2-R | CATGGAAGGGTCCAGATGCCAG | |
| HighGC3-F | AGAGTGGGTCAGAGCAGACA | chr2:167,569,660-167,569,742 |
| HighGC3-R | GGCCTCCCTGTGACTCTTTC | |
| HighGC4-F | GTGCCCTGTTTGTGAGGGTA | chr2:24,272,044-24,272,202 |
| HighGC4-R | CTCTCCCCTCTCAGAGACCC | |
| HighGC5-F | AGGGAACTGCAGACAATGGG | chr3:87,796,637-87,796,726 |
| HighGC5-R | TCACATCTCACTGGCCACAC | |
| HighGC6-F | GGCTGTTCCCCCACTAACTC | chr11:117,268,130-117,268,242 |
| HighGC6-R | CAGACAGGGACAGGATGCAG | |
| HighGC7-F | CCCCAAATAGCAAGGCAAGC | chr11:5,771,081-5,771,208 |
| HighGC7-R | CAGCCCTGCCCTTAGATTCC | |
| HighGC8-F | GAGTTCAGTGGGGTGAGAGC | chr11:61,472,968-61,473,058 |
| HighGC8-R | CACAGCATACACAGCCCCTA | |
| HighGC9-F | GAATGGTGGTCTGTGGGAGG | chr19:4,152,836-4,152,970 |
| HighGC9-R | CCATGGCTGGCAGACTGTAA | |
| | | |
| LowGC1-F | ATCTGATGATGCGATGGTTG | chr1:57,031,228-57,031,316 |
| LowGC1-R | AACGCAAATGACGATTTTCC | |
| LowGC2-F | ACGGGAGTGACTGCATTTTC | chr10:112,815,661-112,815,749 |
| LowGC2-R | CCAGGGAAGCATATTTGAAAG | |
| LowGC3-F | AGGGCCTGAAGGTTTTGTCT | chr11:83,667,373-83,667,452 |
| LowGC3-R | GGGGAATTAACAGCGAGATG | |
| LowGC4-F | CACAACACCAAGCGATAAACA | chr12:58,086,773-58,086,862 |
| LowGC4-R | TTGCAACGTATTGAGAGCAAA | |
| LowGC5-F | GCGTACAGAGATTGCTGTCGTA | chr13:23,610,487-23,610,586 |
| LowGC5-R | TTGTCCTTATCCGCATGTCA | |
| LowGC6-F | ACCTCGAGATTCAGCAAGGA | chr13:89,546,278-89,546,362 |
| LowGC6-R | CACGCACAACAGACATTCAA | |
| LowGC7-F | ATTACAAGCCCGGTCTCCTT | chr14:62,306,729-62,306,824 |
| LowGC7-R | TTGTGGCTTAGCTCATGGTG | |
| LowGC8-F | TTGCCACACCTACTTCAAACC | chr14:118,988,545-118,988,628 |
| LowGC8-R | TCATCGTGATCCTCAATAGCC | |
| LowGC9-F | GAACTTCGAGCCCAGCATT | chr16:94,096,053-94,096,136 |
| LowGC9-R | TCCTAATGGAATCGGTGACA | |
| LowGC10-F | TGGAATTCGCTGTCTAGCAA | chr17:90,563,175-90,563,272 |
| LowGC10-R | CCTTTGACCTTACAGATTTTAACCA | |
| LowGC11-F | TGGTGCCCCCTTATAATCAA | chr6:12,644,244-12,644,339 |
| LowGC11-R | GCAGATGCGGTATTTCATCC | |
| LowGC12-F | GCAGATGAAGCTTCCGAAAA | chr5:123,590,435-123,590,520 |
| LowGC12-R | ACCCAATCCATTCTGTCCTT | |
| LowGC13-F | AGCATGTTTCTTCGAGTCTTG | chr18:26,938,061-26,938,153 |
| LowGC13-R | AGGACAATCATCTGTTCGACAA | |

**SUPPLEMENTARY TABLE 3.** Oligo information for EMSA

| Name | Sequence | GC% | CpG# | Locus (mm9) |
|---|---|---|---|---|
| **HighGC oligos** | | | | |
| HighGC1-F | Biotin-TCTCACTGAAGGTGCGCTAGGGAGATGCTGGGTGGGAAACACGCGGCCGG | 64 | 4 | chr1:184,379,735-184,379,784 |
| HighGC1-R | Biotin-CCGGCCGCGTGTTTCCCACCCAGCATCTCCCTAGCGCACCTTCAGTGAGA | | | |
| HighGC2-F | Biotin-GCTTCCCACTGCTGAGGCCGGTGCCCGCGGAGCTTCTCCCTACGTATTTA | 62 | 4 | chr10:84,595,865-84,595,914 |
| HighGC2-R | Biotin-TAAATACGTAGGGAGAAGCTCCGCGGGCACCGGCCTCAGCAGTGGGAAGC | | | |
| HighGC3-F | Biotin-GAGATCCAGGCGGCCCAAGAGCAGCAGACACCGGAGGCCTGCATGCGCCA | 68 | 3 | chr11:68,343,455-68,343,504 |
| HighGC3-R | Biotin-TGGCGCATGCAGGCCTCCGGTGTCTGCTGCTCTTGGGCCGCCTGGATCTC | | | |
| HighGC4-F | Biotin-TGACTGGAGCCACGCCTCCGGAAGCAGGAATCACATGCCGCGGTAGTTGC | 62 | 4 | chr12:25,544,357-25,544,406 |
| HighGC4-R | Biotin-GCAACTACCGCGGCATGTGATTCCTGCTTCCGGAGGCGTGGCTCCAGTCA | | | |
| HighGC5-F | Biotin-ACCACCCTCGAGGATGGCTTGGGGTCGGGTGGGAAAGTGAGCCTGGCGTG | 66 | 3 | chr13:55,093,651-55,093,700 |
| HighGC5-R | Biotin-CACGCCAGGCTCACTTTCCCACCCGACCCCAAGCCATCCTCGAGGGTGGT | | | |
| HighGC6-F | Biotin-CAGCAGCCACAACAACGAGCCCAGAAGGAGCGGCCTCCTCCTCGGCGGGA | 68 | 4 | chr14:56,331,426-56,331,475 |
| HighGC6-R | Biotin-TCCCGCCGAGGAGGAGGCCGCTCCTTCTGGGCTCGTTGTTGTGGCTGCTG | | | |
| HighGC7-F | Biotin-CTGCACCGCACCTCACAAGTACGCTCGTTCGTCCTCTCCCTCATGCCCAC | 62 | 4 | chr15:25,392,536-25,392,585 |
| HighGC7-R | Biotin-GTGGGCATGAGGGAGAGGACGAACGAGCGTACTTGTGAGGTGCGGTGCAG | | | |
| HighGC8-F | Biotin-CACTCTTCGGTAGCCTGTTCCTGTGGCGGGAGCTGCTGGAGGTACCGCTG | 64 | 3 | chr15:76,165,056-76,165,105 |
| HighGC8-R | Biotin-CAGCGGTACCTCCAGCAGCTCCCGCCACAGGAACAGGCTACCGAAGAGTG | | | |
| **Methylated HighGC Oligos** | | | | |
| mHighGC1-F | Biotin-TCTCACTGAAGGTG$^{m}$**C**GCTAGGGAGATGCTGGGTGGGAAACACGCGGCCGG | 64 | 4 | chr1:184,379,735-184,379,784 |
| mHighGC1-R | Biotin-CCGGCCGCGTGTTTCCCACCCAGCATCTCCCTAG$^{m}$**C**GCACCTTCAGTGAGA | | | |
| mHighGC2-F | Biotin-GCTTCCCACTGCTGAGGCCGGTGCC$^{m}$**C**GCGGAGCTTCTCCCTACGTATTTA | 62 | 4 | chr10:84,595,865-84,595,914 |
| mHighGC2-R | Biotin-TAAATACGTAGGGAGAAGCTCCG$^{m}$**C**GGGCACCGGCCTCAGCAGTGGGAAGC | | | |
| mHighGC3-F | Biotin-GAGATCCAGGCGGCCCAAGAGCAGCAGACAC$^{m}$**C**GGAGGCCTGCATGCGCCA | 68 | 3 | chr11:68,343,455-68,343,504 |
| mHighGC3-R | Biotin-TGGCGCATGCAGGCCTC$^{m}$**C**GGTGTCTGCTGCTCTTGGGCCGCCTGGATCTC | | | |
| mHighGC4-F | Biotin-TGACTGGAGCCACGCCTC$^{m}$**C**GGAAGCAGGAATCACATGCCGCGGTAGTTGC | 62 | 4 | chr12:25,544,357-25,544,406 |
| mHighGC4-R | Biotin-GCAACTACCGCGGCATGTGATTCCTGCTTC$^{m}$**C**GGAGGCGTGGCTCCAGTCA | | | |
| mHighGC5-F | Biotin-ACCACCCTCGAGGATGGCTTGGGGT$^{m}$**C**GGGTGGGAAAGTGAGCCTGGCGTG | 66 | 3 | chr13:55,093,651-55,093,700 |
| mHighGC5-R | Biotin-CACGCCAGGCTCACTTTCCCACC$^{m}$**C**GACCCCAAGCCATCCTCGAGGGTGGT | | | |
| mHighGC6-F | Biotin-CAGCAGCCACAACAA$^{m}$**C**GAGCCCAGAAGGAGCGGCCTCCTCCTCGGCGGGA | 68 | 4 | chr14:56,331,426-56,331,475 |
| mHighGC6-R | Biotin-TCCCGCCGAGGAGGAGGCCGCTCCTTCTGGGCT$^{m}$**C**GTTGTTGTGGCTGCTG | | | |
| mHighGC7-F | Biotin-CTGCACCGCACCTCACAAGTA$^{m}$**C**GCTCGTTCGTCCTCTCCCTCATGCCCAC | 62 | 4 | chr15:25,392,536-25,392,585 |
| mHighGC7-R | Biotin-GTGGGCATGAGGGAGAGGACGAACGAG$^{m}$**C**GTACTTGTGAGGTGCGGTGCAG | | | |
| mHighGC8-F | Biotin-CACTCTTCGGTAGCCTGTTCCTGTGG$^{m}$**C**CGGGAGCTGCTGGAGGTACCGCTG | 64 | 3 | chr15:76,165,056-76,165,105 |
| mHighGC8-R | Biotin-CAGCGGTACCTCCAGCAGCTCC$^{m}$**C**GCCACAGGAACAGGCTACCGAAGAGTG | | | |
| **LowGC** | | | | |
| LowGC1-F | Biotin-ATTATTTCATTTTTTTCTATACGCCGTTAGTAATTTTCGTAGAAAGAACG | 28 | 3 | chr4:53,726,428-53,726,477 |
| LowGC1-R | Biotin-CGTTCTTTCTACGAAAATTACTAACGGCGTATAGAAAAAAATGAAATAAT | | | |
| LowGC2-F | Biotin-TGAAGTAGACTTAGTAAGTTGAGTATCACGTTTCGTTTCGTTTTTATTTT | 30 | 3 | chr16:40,571,836-40,571,885 |

| | | | | |
|---|---|---|---|---|
| LowGC2-R | Biotin-AAAATAAAAACGAAACGAAACGTGATACTCAACTTACTAAGTCTACTTCA | | | |
| LowGC3-F | Biotin-ATAACATCTGTATATATCGATGCGCTTCGGTATCATGTACATGAATATTC | 34 | 3 | chr19:49,142,886-49,142,935 |
| LowGC3-R | Biotin-GAATATTCATGTACATGATACCGAAG CGCATCGATATATACAGATGTTAT | | | |
| LowGC4-F | Biotin-TATAGTAACAAAATAACGATGGCGTGTCGTATCAGAGGAGAAATAAATCT | 34 | 3 | chr16:75,585,018-75,585,067 |
| LowGC4-R | Biotin-AGATTTATTTCTCCTCTGATACGACACGCCATCGTTATTTTGTTACTATA | | | |
| LowGC5-F | Biotin-AGTAGGAGGATGAATACGATTTATAAATATCGCGTATGAAATTCTCAAAG | 32 | 3 | chr3:25,282,230-25,282,279 |
| LowGC5-R | Biotin-CTTTGAGAATTTCATACGCGATATTTATAAATCGTATTCATCCTCCTACT | | | |
| LowGC6-F | Biotin-ACATTTATTTATTTCTTCGATATAAAGTTTATTAGAACCGCCGCTATGTT | 28 | 3 | chr4:110,122,946-110,122,995 |
| LowGC6-R | Biotin-AACATAGCGGCGGTTCTAATAAACTTTATATCGAAGAAATAAATAAATGT | | | |

| **Methylated LowGC Oligos** | | | | |
|---|---|---|---|---|
| mLowGC1-F | Biotin-ATTATTTCATTTTTTTCTATA$^{m}$**C**GCCGTTAGTAATTTTCGTAGAAAGAACG | 28 | 3 | chr4:53,726,428-53,726,477 |
| mLowGC1-R | Biotin-CGTTCTTTCTACGAAAATTACTAACGG$^{m}$**C**GTATAGAAAAAAATGAAATAAT | | | |
| mLowGC2-F | Biotin-TGAAGTAGACTTAGTAAGTTGAGTATCA$^{m}$**C**GTTTCGTTTCGTTTTTATTTT | 30 | 3 | chr16:40,571,836-40,571,885 |
| mLowGC2-R | Biotin-AAAATAAAAACGAAACGAAA$^{m}$**C**GTGATACTCAACTTACTAAGTCTACTTCA | | | |
| mLowGC3-F | Biotin-ATAACATCTGTATATATCGATG$^{m}$**C**GCTTCGGTATCATGTACATGAATATTC | 34 | 3 | chr19:49,142,886-49,142,935 |
| mLowGC3-R | Biotin-GAATATTCATGTACATGATACCGAAG$^{m}$**C**GCATCGATATATACAGATGTTAT | | | |
| mLowGC4-F | Biotin-TATAGTAACAAAATAACGATGG$^{m}$**C**GTGTCGTATCAGAGGAGAAATAAATCT | 34 | 3 | chr16:75,585,018-75,585,067 |
| mLowGC4-R | Biotin-AGATTTATTTCTCCTCTGATACGACA$^{m}$**C**GCCATCGTTATTTTGTTACTATA | | | |
| mLowGC5-F | Biotin-AGTAGGAGGATGAATA$^{m}$**C**GATTTATAAATATCGCGTATGAAATTCTCAAAG | 32 | 3 | chr3:25,282,230-25,282,279 |
| mLowGC5-R | Biotin-CTTTGAGAATTTCATACGCGATATTTATAAAT$^{m}$**C**GTATTCATCCTCCTACT | | | |
| mLowGC6-F | Biotin-ACATTTATTTATTTCTT$^{m}$**C**GATATAAAGTTTATTAGAACCGCCGCTATGTT | 28 | 3 | chr4:110,122,946-110,122,995 |
| mLowGC6-R | Biotin-AACATAGCGGCGGTTCTAATAAACTTTATAT$^{m}$**C**GAAGAAATAAATAAATGT | | | |

## SUPPLEMENTARY METHODS

### Electrophoretic Mobility Shift Assay (EMSA)

Production of recombinant *Mecp2* isoforms 1 and 2 was performed in E. coli by cloning the appropriate cDNAs from expression plasmids (OriGene # MR207745 and #MR226839) into pT7-7 using the restriction enzymes Nde I and Eco RI. pT7-Mecp2 plasmids were sequence validated and transformed into BL21 star cells for protein expression. Following IPTG induction, the soluble fraction of the bacterial lysate was purified via desalting (Hi Prep 26/10 desalting column) followed by ion exchange chromatography on Source Q using an AKTA FPLC (GE Healthcare, Piscataway, NJ). Buffers used are 10 mM Tris pH 7.5, 1 mM EDTA, 10% glycerol, with and without 1 M NaCl. Column fractions were screened by SDS-PAGE, the peak fraction identified, and further validated by western blotting (Supplementary figure 10a). Activity of MeCP2 was examined and quantified by EMSA using methylated double stranded oligos containing methylated CpGs (see Supplementary Table 3). After determining activity, MeCP2 (isoform2) was tested for binding activity against 6 low GC oligos (50 bp in length with 28-34% GC) and 8 high GC oligos (50 bp in length with 62-70% GC). All oligo sequences were selected from the mm9 mouse genome (see Supplementary Table 3). Both forward and reverse oligos were modified with 5'biotin. After complementary annealing, double stranded oligos were used for EMSA. Corresponding methylated oligos, each containing one symmetrically methylated CpG, were also evaluated.

An equal amount of MeCP2 was used in all binding reactions. The binding condition was: 10 mM Tris, 75 mM KCl, 1 mM DTT, 1% glycerol, 20 fmol of biotinylated oligos with 50 ng $\mu l^{-1}$ of poly dA:dT as competitor (Invivogen, Cat#NC0380747) in 20 μl reaction. After 30 min of binding, DNA/MeCP2 complexes were electrophoresed on 6% polyacrylamide TBE gel. Hybond-N+ nylon was used to immobilize biotinylated oligos. Detection of biotinylated oligos was done using LightShift Chemiluminescent EMSA kit (Thermo Scientific). Collection of images and quantification of chemiluminescent signals were done using ChemDoc-It2 Imager by UVP.

### Cross-correlation Analysis

The auto- and cross-correlation functions for ChIP-seq and MNase-seq data sets were calculated using the strand-specific counts of mapped reads at single base pair resolution. Because most base pairs have no reads

mapped to them, the signal is under-sampled at this resolution and the correlations are thus low. Furthermore, the different data sets differed in sequencing depth, making it difficult to compare the cross- and auto-correlations between sets. To address these problems, we modeled the number of reads mapping to track $a$ at position $i = 1 \dots L$ as $X_{a,i} \sim \mathrm{Pois}(\lambda_{a,i})$, where the sampling rate $\lambda_{a,i}$ is the signal, the statistical properties of which we would like to know. However, the values $\lambda_{a,i}$ are unknown, and we model them as identical random variables that become independent for large genomic separation. The population correlation of the counts is then proportional to that of the sampling rates:

$$\rho(X_{1,i}, X_{2,i+\delta}) = \rho(\lambda_{1,i}, \lambda_{2,i+\delta}) \prod_{a=1,2} \sqrt{\frac{\mathrm{Var}[\lambda_a]}{\mathrm{Var}[\lambda_a] + \mathrm{E}[\lambda_a]}}$$

To understand how varying the sequencing depth changes this correlations, consider the reparametrization $\lambda_{a,i} = \alpha_a \, \Lambda_{a,i}$ where $\alpha_a$ is the mean sequencing depth and $\Lambda_{a,i}$ is normalized sampling rate with $\mathrm{E}[\Lambda_a] = 1$. Then

$$\rho(X_{1,i}, X_{2,i+\delta}) = \rho(\Lambda_{1,i}, \Lambda_{2,i+\delta}) \prod_{a=1,2} \sqrt{\frac{\alpha_a \mathrm{Var}[\Lambda_a]}{\alpha_a \mathrm{Var}[\Lambda_a] + 1}}.$$

When the sequencing depth is much smaller than one, this formula gives $\rho(X_{1,i}, X_{2,i+\delta}) \ll \rho(\Lambda_{1,i}, \Lambda_{2,i+\delta})$<1, consistent with the low correlations observed at single base pair resolution. While it is necessary to know $\mathrm{Var}[\Lambda_a]$ to calculate $\rho(\Lambda_{1,i}, \Lambda_{2,i+\delta})$ from $\rho(X_{1,i}, X_{2,i+\delta})$, the correlation of the counts scales like $\sqrt{\alpha_1 \alpha_2}$ for $\alpha_a \ll 1/\mathrm{Var}[\Lambda_a]$, and this scaling is useful for relating correlations observed at different sequencing depths. To see how the sample auto- and cross-correlation change with the sequencing depth, first note that $\overline{X_a} \cong \mathrm{E}[X_{a,i}]$ for $L \gg 1$. Furthermore, the sample cross-covariance is $\sum_j \frac{1}{L-1}(X_{1,j} - \overline{X_1})(X_{2,j+\delta} - \overline{X_2}) \cong \mathrm{Cov}[X_{1,i}, X_{2,i+\delta}]$ for $L \gg 1$ and similarly for the sample variance. Thus, sample cross-correlation $r(X_1, X_2; \delta)$ also scales like $\sqrt{\alpha_1 \alpha_2}$ for $\alpha_a \ll \frac{1}{Var[\Lambda_a]}$. Because the simple power scaling saturates around $\alpha_a \sim 1/\mathrm{Var}[\Lambda_a]$, we chose to present the normalized cross- and auto-correlations at the common sequencing depth $\alpha_{\mathrm{norm}} = \frac{1}{4}$. Thus, the normalized sample correlation is

$$r_{\mathrm{norm}}(X_1, X_2; \delta, \alpha_{\mathrm{norm}}) = r(X_1, X_2; \delta) \prod_{a=1,2} \sqrt{\frac{\alpha_{\mathrm{norm}}}{\bar{X}_a}}.$$

Note that while the choice of absolute normalization $\alpha_{\mathrm{norm}}$ is somewhat arbitrary, this normalization procedure allows for rigorous comparison of signals with different sequencing depth. Also note that the assumption that $X_{a,i}$ is Poisson distributed can be significantly relaxed; if $X_a$ is distributed according to some distribution parameterized such that $\mathrm{E}[X_i] = \mu_i$, then

$$\rho(X_1, X_2) = \rho(\mu_1, \mu_2) \sqrt{\prod_{i=1,2} \frac{\mathrm{Var}[\mu_a]}{\mathrm{Var}[\mu_a] + \mathrm{E}[\mathrm{Var}[X_a|\mu_a]]}}$$

The above procedure thus works if $\mathrm{Var}[X_i|\mu_i] = \mu_i$ when $\mu_i \ll 1$, a condition that is true both for the Bernouli and for the Negative Binomial distributions.

## SUPPLEMENTARY REFERENCES


1 Baubec, T., Ivanek, R., Lienert, F. & Schubeler, D. Methylation-dependent and -independent genomic targeting principles of the MBD protein family. *Cell* **153**, 480-492, doi:10.1016/j.cell.2013.03.011 (2013).

2 Gabel, H. W. *et al.* Disruption of DNA-methylation-dependent long gene repression in Rett syndrome. *Nature* (2015).

3 Chen, L. *et al.* MeCP2 binds to non-CG methylated DNA as neurons mature, influencing transcription and the timing of onset for Rett syndrome. *Proceedings of the National Academy of Sciences* **112**, 5509-5514 (2015).

4 Lister, R. *et al.* Global epigenomic reconfiguration during mammalian brain development. *Science (New York, N.Y.)* **341**, 1237905 (2013).

5 Stadler, M. B. *et al.* DNA-binding factors shape the mouse methylome at distal regulatory regions. *Nature* (2011).

6 Skene, P. J. *et al.* Neuronal MeCP2 is expressed at near histone-octamer levels and globally alters the chromatin state. *Molecular cell* **37**, 457-468, doi:10.1016/j.molcel.2010.01.030 (2010).

7 Clowney, E. J. *et al.* High-throughput mapping of the promoters of the mouse olfactory receptor genes reveals a new type of mammalian promoter and provides insight into olfactory receptor gene regulation. *Genome research* **21**, 1249-1259 (2011).
